# Dimension-reduction simplifies the analysis of signal crosstalk in a bacterial quorum sensing pathway

**DOI:** 10.1038/s41598-021-99169-0

**Published:** 2021-10-05

**Authors:** Taylor Miller, Keval Patel, Coralis Rodriguez, Eric V. Stabb, Stephen J. Hagen

**Affiliations:** 1 Physics Department, University of Florida, Gainesville, FL 32611-8440 USA; 2 Department of Microbiology, University of Georgia, Athens, GA 30602 USA; 3 Department of Biological Sciences, University of Illinois, Chicago, IL 60607 USA

**Keywords:** Computational biophysics, Biological physics, Microbiology, Applied microbiology, Bacteria, Environmental microbiology

## Abstract

Many pheromone sensing bacteria produce and detect more than one chemically distinct signal, or autoinducer. The pathways that detect these signals are typically noisy and interlocked through crosstalk and feedback. As a result, the sensing response of individual cells is described by statistical distributions that change under different combinations of signal inputs. Here we examine how signal crosstalk reshapes this response. We measure how combinations of two homoserine lactone (HSL) input signals alter the statistical distributions of individual cell responses in the AinS/R- and LuxI/R-controlled branches of the *Vibrio fischeri* bioluminescence pathway. We find that, while the distributions of pathway activation in individual cells vary in complex fashion with environmental conditions, these changes have a low-dimensional representation. For both the AinS/R and LuxI/R branches, the distribution of individual cell responses to mixtures of the two HSLs is effectively one-dimensional, so that a single tuning parameter can capture the full range of variability in the distributions. Combinations of crosstalking HSL signals extend the range of responses for each branch of the circuit, so that signals in combination allow population-wide distributions that are not available under a single HSL input. Dimension reduction also simplifies the problem of identifying the HSL conditions to which the pathways and their outputs are most sensitive. A comparison of the maximum sensitivity HSL conditions to actual HSL levels measured during culture growth indicates that the AinS/R and LuxI/R branches lack sensitivity to population density except during the very earliest and latest stages of growth respectively.

## Introduction

Bacteria communicate with each other and sense their environment by exchanging small diffusible pheromone molecules known as autoinducers. Although this signaling mechanism was initially interpreted as a means of triggering phenotypic changes in response to population density, pheromone sensing networks often have elaborate architecture that indicates their function is more complex than simply sensing population or “quorum”^1–3^. These pathways respond not only to extracellular concentrations of autoinducer, but also to other environmental cues, metabolic parameters, and signals from other species^4,5^. In addition, many bacteria synthesize and sense more than one chemically distinct autoinducer^6,7^. They detect these signals using multiple receptor pathways in interlocked configurations that often involve feedback, and use them to regulate multiple phenotypes. The question of how such configurations extend the sensing capabilities of these networks remains of great interest^8^.

These sensing pathways can be subject to crosstalk between the different signals and detection mechanisms. In Gram-negative bacteria, the autoinducers are often N-acylated homoserine lactones (HSLs), which may be produced or detected with different degrees of specificity^5,8^. One form of crosstalk occurs when receptors in one branch of a pathway interact with non-cognate HSLs produced by another pathway in the same organism. Crosstalk may also occur in system architectures where receptors for several different autoinducers drive the same downstream outputs, so that information arriving from different autoinducers is merged into a common output from a regulatory pathway.

One obstacle to understanding how a cell's sensing functions benefit from the presence of multiple, interacting pathways is the heterogeneity of gene expression: given a well-defined concentration of autoinducer in the environment, the response of individual cells exhibits a statistical distribution^9^. For example, even when *Vibrio fischeri* are provided precisely-controlled autoinducer concentrations, the response of the pheromone controlled bioluminescence pathway may differ by an order of magnitude from one cell to another^10^. Although such variability may provide a fitness benefit at the population level^11^, it makes the behavior of an individual cell an unreliable indicator of the input signals it is receiving. In addition, a population-averaged measurement of behavior cannot capture the diversity of individual cell behaviors that occur in response to a signal input. This raises the question of how crosstalk between two interacting pathways affects the sensitivity of those pathways and the range and statistical distributions of the outputs. It also raises the question of how subtle effects of crosstalk can be detected or characterized. For example it is conceivable that crosstalk could modulate the sensitivity of a pathway to environmental cues, not by changing the mean response of the population, but through changes in the distribution of responses, which affect phenotypic variability and bet hedging^12^. To understand how crosstalk affects sensing, it is necessary to look beyond average population responses and determine whether and how multiple signals reshape the statistical distribution of individual cell responses.

Here we have measured how crosstalking HSL signals affect the heterogeneous response of two branches of the *V. fischeri* pheromone sensing network. The AinS/R and LuxI/R pathways in *V. fischeri* comprise two distinct but interacting, sensing pathways that are important to colonization of the microbe's animal host. They are linked in a generally sequential fashion^13^, but they also interact through several different crosstalk mechanisms.

**Figure 1:**
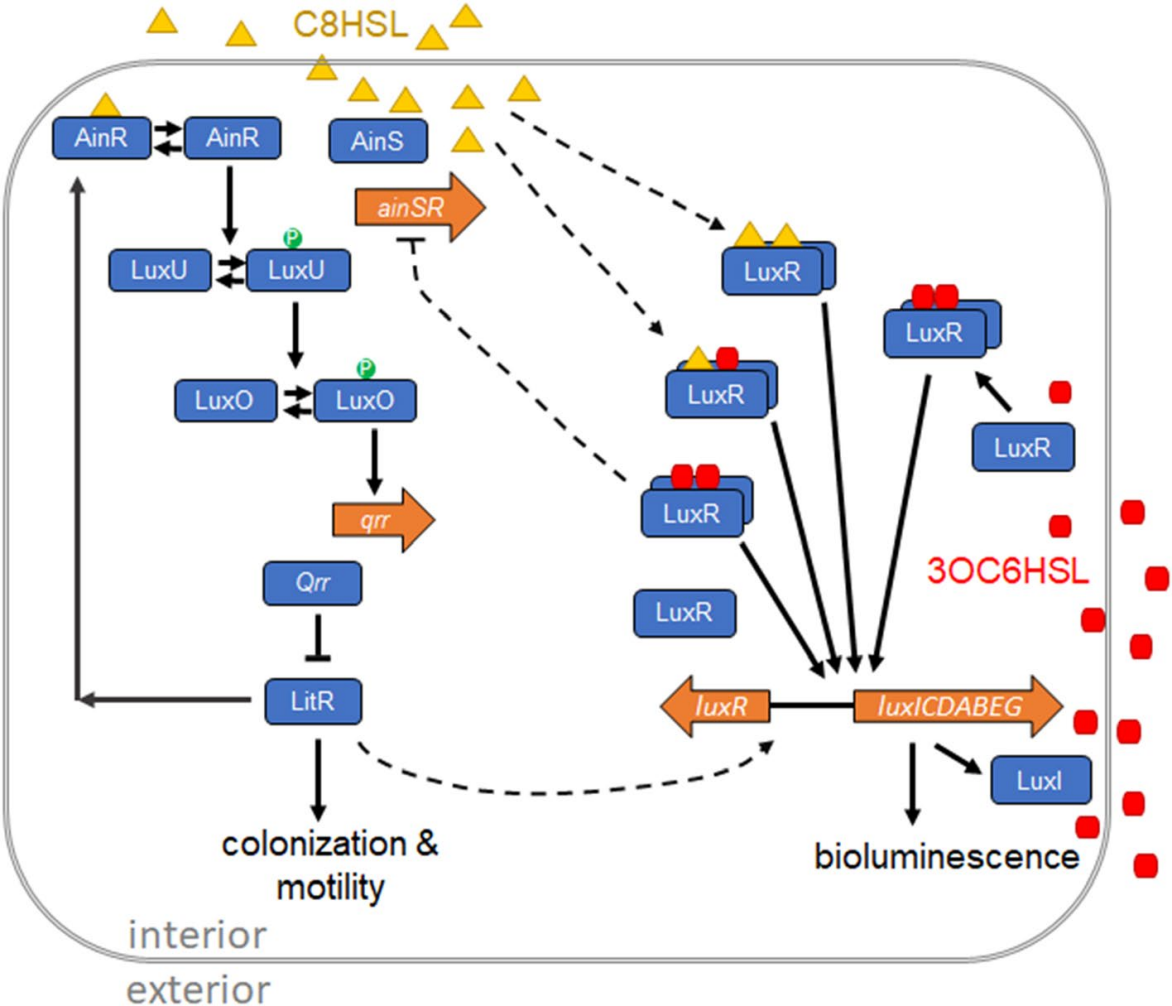
Schematic of the *Vibrio fischeri* AinS/R and LuxI/R pheromone sensing systems and their crosstalk*.* (AI2 signaling via LuxP/Q is not shown.) AinS and AinR are the synthase and cognate receptor respectively for C8HSL. At low extracellular C8HSL concentrations, AinR drives the LuxU/LuxO phosphorylation cascade which stimulates transcription of the regulatory RNA Qrr, which posttranscriptionally inhibits expression of *litR*. At high C8HSL concentrations, LuxU and LuxO are dephosphorylated and *qrr* transcription is suppressed, allowing production of LitR. LitR controls colonization and motility phenotypes. LuxR is the intracellular receptor for the autoinducer 3OC6HSL, produced by LuxI. LuxR interacts with 3OC6HSL to form a dimeric transcriptional activator complex that binds the *lux box* site, which controls expression of the *lux* operon, *luxICDABEG.* The *lux* operon controls synthesis of the bacterial luciferase and its substrate, which are responsible for bioluminescence. The AinR/S and LuxI/R pathways are subject to crosstalk through several mechanisms, which are indicated by dashed curves: C8HSL interacts with LuxR both alone and in combination with 3OC6HSL; 3OC6HSL influences *ainSR* transcription through a LuxR-dependent interaction; LitR activates *luxR* transcription.

Figure 1 gives an overview of these mechanisms^14^. The AinS/R branch of the system controls expression of *litR*. It synthesizes (via AinS) and detects (via AinR) the autoinducer *N*-octanoyl-l-homoserine lactone (C8HSL). When C8HSL is absent or extremely dilute, AinR phosphorylates LuxU, which phosphorylates LuxO, which in turn activates transcription of *qrr1*. The sRNA Qrr1 represses *litR,* which encodes the master regulator LitR. At higher C8HSL concentrations AinR dephosphorylates LuxU, the phosphorylation cascade is reversed, and LitR production increases. LitR has a broad regulon that affects colonization of the symbiotic host, flagellar motility and chemotaxis, exopolysaccharide production, and other factors^15–17^.

LitR also activates *luxR,* which encodes the intracellular receptor LuxR of the LuxI/R branch of the system. LuxI/R controls expression of the *lux* operon, *luxICDABEG,* by synthesizing (via LuxI) and detecting (via LuxR) the autoinducer N-(3-oxohexanoyl)-*L-*homoserine lactone (3OC6HSL). The *lux* operon is responsible for both 3OC6HSL production (LuxI) and bioluminescence (LuxCDABEG)^14^.

*V. fischeri* also detects a third autoinducer, AI2, through the LuxS/P/Q detection system which also feeds into *qrr*. However, under the conditions that have been tested AI2 has only a modest effect on colonization and luminescence^18^. We do not consider it further in this study.

One mechanism of crosstalk in this system is that AinS/R stimulates the LuxI/R branch by upregulating *luxR* via LitR*.* Therefore C8HSL detected by the AinS/R system sensitizes the pathway that detects 3OC6HSL. Another mechanism occurs via the receptor LuxR, which can form the transcriptional activator for *lux* through binding of either C8HSL or 3OC6HSL. Different combinations of these autoinducers activate *lux* with different effectiveness^19^*.* A third mechanism arises through 3OC6HSL, which acts with LuxR to repress *ainSR*, apparently by binding upstream of its promoter, hence indirectly suppressing *qrr*^20,21^.

The presence of these crosstalk mechanisms suggests that combinations of HSLs may have complex effects on the response of each branch. The positive feedback that exists in both systems could accentuate these interactions. If mixtures of the two HSLs allow fine tuning of the joint response, they could enable additional modes or levels of activation during colonization of the host animal. Several prior studies have explored the effects of multiple driving inputs on statistical distributions of quorum-regulated gene expression, typically by characterizing the mean and variance in expression of a reporter^22–24^. However, mean and variance may be insufficient to describe changes in these statistical distributions, especially as the distributions need not follow any known form. The relevant parameters, or even the number of relevant parameters, that describe the distribution are not necessarily known. Common parameters like mean and variance may be correlated or may fail to capture fundamental changes in the shape or asymmetry of a distribution, such as a transition to bimodality.

Here we use a data-based, dimension-reduction approach to explore how individual cell behavior changes when different HSLs are present in combination. Dimension reduction allows easier visualization of complex datasets and facilitates interpretation and modeling of that data^25^. Using promoter activity of *qrr* and *luxI* as readouts, we collected histograms of individual-cell activity in the AinS/R and LuxI/R branches respectively, for different combinations of the two HSLs. Applying principal component analysis (PCA) to these histograms allows us to assess whether the two-dimensional (two-HSL) space of signal inputs generates a similar, multidimensional space of possible output distributions of each branch. The dimensional-reduction provided by PCA, and especially nonlinear PCA^26^, facilitates clear visualization of how noisy cell behavior is shaped by combinations of input signals. This in turn allows us to map the HSL concentrations for which the distribution of *qrr* and *luxI* reporter activity is most sensitive or responsive, providing insight into the environmental inputs that the *V. fischeri* sensing system appears attuned to detect.

## Methods

### Reporter strains

Table 1 describes the *Vibrio fischeri* strains and plasmids. All strains were derived from ES114, a wild-type isolate from the light organ of a natural squid host^27^. The *lux-* and *qrr-*reporting strains carry an *ainS* deletion in addition to a frameshift mutation in *luxI*, leaving them unable to synthesize either 3OC6HSL or C8HSL. The *qrr* reporting strain was NL63 pTM268. It was created by inserting pTM268^28^, which carries a P*qrr-gfp* fusion, into the synthase-deficient strain NL63^20^ through triparental mating using helper strain CC118 λpir pEVS104^29,30^. Transconjugants were selected for antibiotic resistance on 1.5 wt% agar plates.

**Table 1:** Strains used in this study.

Strain or plasmid	Relevant characteristic or sequence	Source or reference
*V. fischeri* ES114	Wild type	Boettcher and Ruby, 1990
*V. fischeri* NL63	ES114 Δ*ainS luxI*	Kimbrough et al.,2013
*V. fischeri* NL63 pTM268	NL63 + pTM268	This Study
*V. fischeri* DC59	ES114 Δ*ainS* Δ*luxI-luxR, mutant luxR*^*B*^*(MJ1-T33A, R67M, S116A, M135I),* P_*luxI*_-*luxDCABEG, lonA:Tn*	This Study
*V. fischeri* DC60	ES114 Δ*ainS* Δ*luxI-luxR, luxR*^*ES114*^*,* P_*luxI*_-*luxDCABEG, lonA:Tn*	This Study
*V. fischeri* CR11	ES114 Δ*ainS luxI* P_*luxI*_-*gfp,*	This Study
*E. coli* CC118*λpir*	Δ(*ara-leu*) *araD* Δ*lac74 galE galK phoA20 thi-1 rpsE rpsB argE*(Am) *recA λpir*	^30^
pTM268	P*qrr1*-*gfp*, mCherry, pES213, R6K *oriT*RP4 *camR*	^28^

The *lux* reporting strain was CR11, which in addition to lacking HSL synthesis also carries a chromosomal *gfp* reporter inserted into the *lux* operon between the mutant *luxI* allele and *luxC*.

To measure HSL concentrations in growing cultures of ES114 we used reporter strains DC59 and DC60. Both strains lack the ability to synthesize C8HSL (due to *ainS* deletion) and 3OC6HSL (due to a frameshift in *luxI*). The two strains were derived (by addition of a transposon insertion in *lonA*) from the modified LuxR strains DC22 and DJ101, which express *luxR* from a constitutive promoter and luminesce strongly in response to C8HSL or 3OC6HSL respectively^19^. DC59 expresses *luxR*^B^, encoding a LuxR protein that more strongly activates luminescence in the presence of C8HSL^31^. DC60 carries the normal ES114 *luxR* that responds preferentially to 3OC6HSL. HSL responses of these strains are shown in^19^.

### Growth conditions and bulk cultures

Overnight cultures were grown in LBS medium^32^ and then diluted 1:50 into fresh LBS and grown to OD (600 nm) = 0.4–0.6. Cells were then washed twice and resuspended in SWTO medium^33^.

Stock solutions of the HSLs (Cayman Chemical, C8HSL CAS 147852-84-4, 3OC6HSL CAS 76924-95-3, Santa Cruz Biotech, C7HSL, CAS 177158-20-2) were prepared in DMSO and were diluted at least 1000 × into SWTO to create 1 µM and 1 nM stocks for further dilution into live cultures.

We measured *lux* and *qrr* promoter activity in response to 3OC6HSL and C8HSL using bulk fluorescence (in a 96 well plate) and individual cell fluorescence microscopy on the *gfp* reporter strains CR11 (*lux* reporter) and NL63 pTM268 (*qrr* reporter*)*. Cells were washed and then diluted 20 × into solutions of SWTO that contained known concentrations of 3OC6HSL and C8HSL. For both well-plate and individual cell studies, the *qrr* reporting strain was studied for a 7 × 7 grid of HSL concentrations: C8HSL = 0, 7, 20, 50, 100, 200, 1000 pM and 3OC6HSL = 0, 7, 15, 20, 50, 100, 500 nM. The *lux* reporting strain was studied for a 7 × 8 grid of HSL concentrations: C8HSL = 0, 10, 20, 50, 100, 200, 500 nM and 3OC6HSL = 0, 10, 20, 30, 50, 100, 200, 500 nM.

### Single-cell fluorescence microscopy

To prepare samples for single-cell fluorescence microscopy, cultures (each 800 μl) were grown in duplicate for each of 49 (*qrr* reporter) or 56 (*lux* reporter) HSL conditions in a plate reader (BioTek Synergy 2) at 25 °C with medium shaking. Fluorescence and optical density of one member of each duplicate pair was monitored until the culture reached OD (600 nm) = 1.1. Then a sample from the second duplicate was diluted 1:20 into artificial seawater and 2 μl was deposited onto an agarose pad for microscopy. Agarose pads (1.5 wt%) were prepared by pouring warm 1.5 wt% agarose in artificial seawater onto a cover slip and then cooling, detaching the pad from the slide, inverting it, and storing in a humid enclosure^34^.

We collected phase contrast and fluorescence images of the reporting cells on the agarose pads at 20 × using an inverted microscope (Nikon Ti2 Eclipse) with a cooled CMOS camera (Andor Zyla 4.2). CR11 (*lux*) was imaged in green fluorescence only (EGFP filter). NL63 pTM268 (*qrr*) was imaged in both red (Texas Red filter) and green (EGFP filter) fluorescence.

Phase and fluorescence microscopy images were analyzed using a custom Matlab code that identified the location of each cell within a 16 × 16 pixel region by fitting the phase contrast image of that region with a polynomial threshold function. The fluorescence of the cell was then extracted by summing the fluorescence in the identified pixels and subtracting a background signal estimated from the remainder of the 16 × 16 pixel region. This analysis produced the cell location, pixel area, and green (and/or red) fluorescence intensity of each cell as well as an error estimate. We typically analyzed ~ 400 cells per image and excluded fewer than one percent from further analysis, owing to high fit error (e.g. irregular shape) or extremely aberrant fluorescence. Individual cell fluorescence raw values and cell sizes (area in pixels) are in the supplemental data files*SingleCell_lux.xlsx* and *SingleCell_qrr.xlsx*.

The NL63 pTM268 (*qrr* reporting) strain carried a green (*qrr* reporting) fluorescent reporter in addition to a red (*constitutive)* fluorescent reporter as control. In fact we found no significant correlation between the green and red signals, except at HSL conditions where the green fluorescence was very weak. Under that condition both green and red fluorescence correlated more closely with cell area than with each other (Supplementary Figure S1). This finding indicated that the cell size was a more important correlate of the green fluorescence than was the *rfp* expression level. Therefore we removed this correlate from the green fluorescence data by subtracting a small correction, proportional to the cell area, from all green fluorescence values, as shown in Supplemental Figure S2, prior to further analysis.

### PCA dimension reduction of histograms

We measured the statistical distribution of *qrr* and *luxI* reporter activity, in green fluorescence units, for each HSL input condition (Supplemental Figures S3 and S4). To analyze these distributions, we first aggregated (for each reporter strain) all the individual cell fluorescence values observed over all HSL conditions. We then found the locations and widths for 20 fluorescence bins such that all bins were equally populated in this aggregate of all data (one reporter, all HSL conditions). Using these bins we then calculated the histogram of single-cell fluorescence for that reporter at each HSL condition. The histograms and bin properties are shown as Supplemental Figures S5, S6 and S7.

Each histogram can be expressed as a set of 20 probabilities *p*_*i*_ where

$$\sum_{i=1}^{20}{p}_{i}=1$$

and *p*_i_ is the probability that the fluorescence of a cell lies within bin *i*, for *i* = 1–20. For further analysis, rather than working with the *p*_*i*_ we analyzed ${q}_{i}=\sqrt{{p}_{i}}$. This is because

$$\sum_{i=1}^{20}{q}_{i}^{2}=1$$

defines a sphere of unit radius in the 20-dimensional space of the *q*_i_. For a given reporter strain, the full histogram of single-cell data obtained under any HSL condition can then be represented by one point on the surface of 20-dimensional sphere. On this sphere the distance between two points is a useful measure of the difference between the underlying histograms: the Euclidean (straight line) distance between two points is the so-called chord distance between the corresponding histograms^35^, while the arc distance between any two points on the sphere is the information-geometric distance between the histograms^36^. With the transformation from *p* to *q,* the Euclidean distances between points in the Cartesian coordinate system discussed below are measures of meaningful differences between the measured histograms that are represented by those points.

To determine the underlying dimensionality of the set of histograms, we applied a hierarchical nonlinear principal component analysis (*h*)-NLPCA^37^. Standard linear PCA represents the dataset in terms of a series of linear components, which are rank-ordered from largest to smallest by their ability to capture the variance in the dataset. (*h*)-NLPCA extends PCA by identifying a hierarchically ranked set of *n* nonlinear components that can explain the variance in the data. It is hierarchical in the sense that the first *n* components provide the best possible *n-*component representation of the dataset (“scalability”), while the *i*th component of an *n-*component representation does not change if *n* is increased (“stability”). (*h*)-NLPCA is implemented using an autoassociative neural network in which the *n* components form a bottleneck between two hidden layers and the input/output layers, with nonlinear couplings between layers. With *n* components we constructed the neural network in a 20:10:*n*:10:20 configuration, so that the input and output layers each have 20 components (matching the number of bins in the histogrammed data) and the hidden layers each contain 10 components. Varying the number of components in the hidden layers between 5 and 20 had no significant effect on the results.

NLPCA was implemented using the autoassociative neural network (autoencoder) that is available in Matlab^26^ using a weight decay coefficient 0.001 to prevent overfitting. The output of the analysis includes the linear PCA components (scores and loadings) as well as values of the *n* underlying coordinates found from the nonlinear analysis: Each experimentally measured histogram is represented by one point in the space of the linear PCA components, and the analysis identifies an *n-*dimensional subspace in which those points are embedded. For both the *qrr* and *lux* reporter data, we ran the analysis to *n* = 5, allowing the nonlinear subspace to have up to 5 dimensions.

### Measurement of HSL concentrations during culture growth

We measured 3OC6HSL and C8HSL production by strain ES114 growing in SWTO to OD (600 nm) = 1.5 while withdrawing samples periodically. We then used the DC59 and DC60 strains to test the HSL concentration of the extracted samples.

DC59 and DC60 response were calibrated by measuring OD(600 nm) and bioluminescence of DC59 and DC60 growing in a 96 well-plate in a grid of 3OC6HSL (0–200 nM) and C8HSL (0–800 nM) concentrations. These samples were grown to OD (600 nm) 0.8 at 25 °C with medium shaking, and the bioluminescence at OD 0.5 was recorded.

We then diluted an overnight culture of ES114 100 × into fresh SWTO. The flask was shaken at 25 °C and 600 μL samples were periodically removed until OD600 nm = 1.2. Samples were pelletized and the supernatant was filter sterilized. HSL was extracted from the supernatant by mixing equal volumes of ethyl acetate (with 0.1% acetic acid) with the supernatant and vortexing, then allowing the organic and aqueous phases to separate. The organic phase was collected and dried, and the residue was then dissolved in 225 μl SWTO and added to wells containing growing DC59 or DC60. Similar results were obtained when the experiment was repeated without organic solvent extraction. In that case the filter sterilized supernatants were mixed at 1:1 volume ratio with SWTO that contained a reporter strain at OD 0.3–0.4. These samples were placed in a well plate and the luminescence at 0.5 OD was recorded.

## Results

### Well plate assays

**Figure 2:**
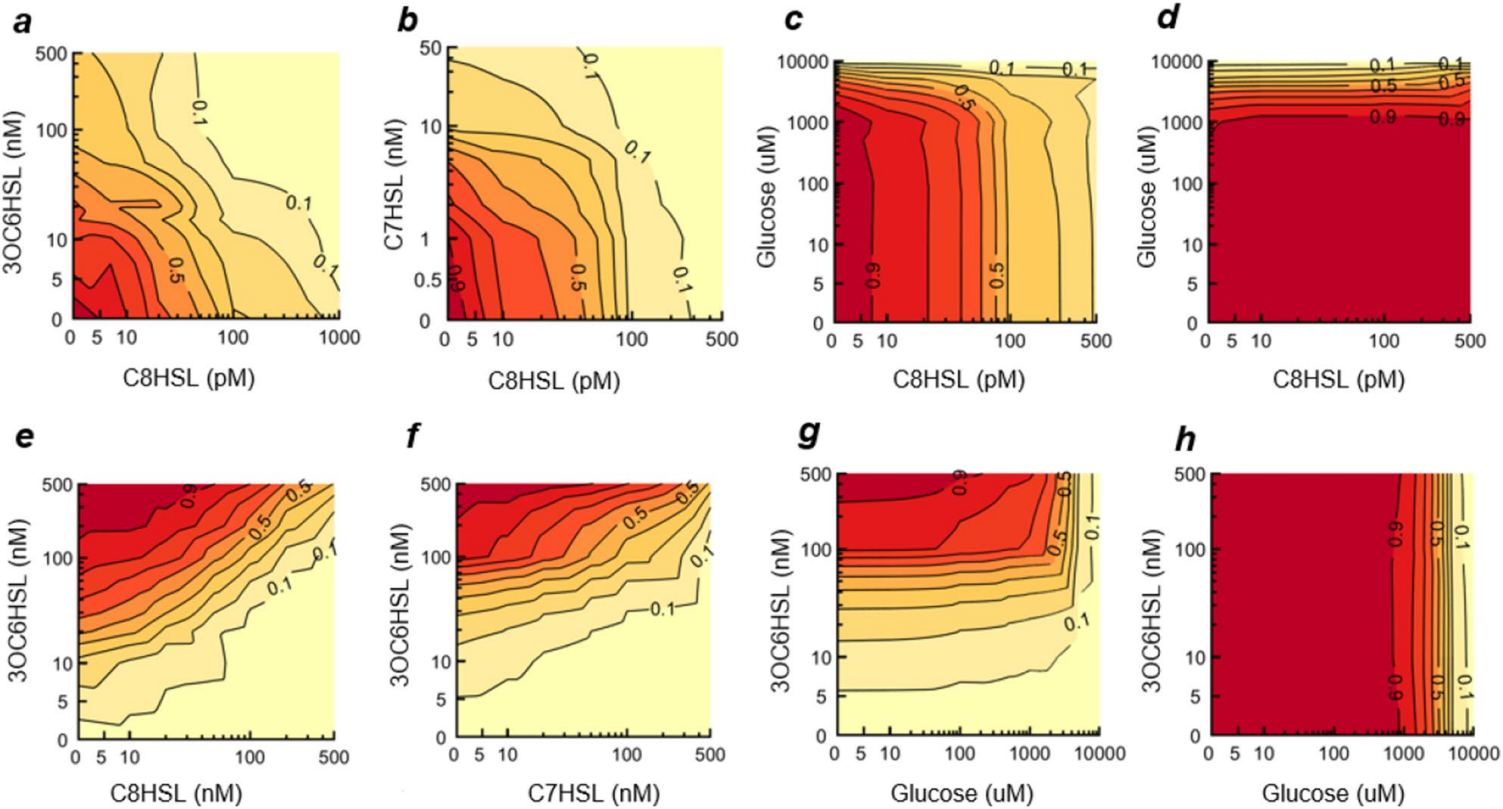
Population-average response of AinR/S and LuxI/R sensing systems. The mean (population-averaged) response of the *qrr* and *lux* reporters to environmental cues is measured using a well-plate reader. Contours show GFP fluorescence and growth behavior measured in bulk cultures for *qrr* and *lux* reporting strains under different combinations of HSL and glucose inputs. For the *qrr* reporter, contour plots show mean fluorescence in response to combinations of **(a)** C8HSL and 3OC6HSL, **(b)** C8HSL and C7HSL, and **(c)** C8HSL and glucose; **(d)** shows optical density (600 nm) following 10 h growth in C8HSL with glucose. For the *lux* reporting strain, contour plots show mean response to **(e)** 3OC6HSL and C8HSL, **(f)** 3OC6HSL and C7HSL, and **(g)** 3OC6HSL and glucose; **(h)** shows optical density (600 nm) following 10 h growth in 3OC6HSL and glucose. The labels on the contours are normalized relative to the maximum observed data value. Axis scales are linear below 10 concentration units and logarithmic above 10 units^48^.

We first used well-plate (bulk culture) measurements to confirm that the population-averaged response of both the *qrr* and *lux* reporters is consistent with prior reports, and to identify some of the environmental inputs that affect it. Figure 2 shows how the *qrr* and *lux* reporters respond to combinations of C8HSL and 3OC6HSL as well as glucose and the non-cognate C7HSL. Consistent with^20^, both C8HSL and C7HSL suppress mean *qrr* activity, with C8HSL effective at picomolar concentrations. The *lux* reporter is activated by 3OC6HSL and inhibited by C8HSL as expected^19^. Similarly 3OC6HSL and C7HSL have generally opposite effects on *lux* activity, with about 100 nM C7HSL sufficient to inhibit the *lux* response to 3OC6HSL. Glucose suppresses activity in both reporters, at roughly the same concentration at which it impacts growth yield in presence of either C8HSL or 3OC6HSL (Fig. 2).

### Analyzing histograms of individual cell reporter activity

**Figure 3:**
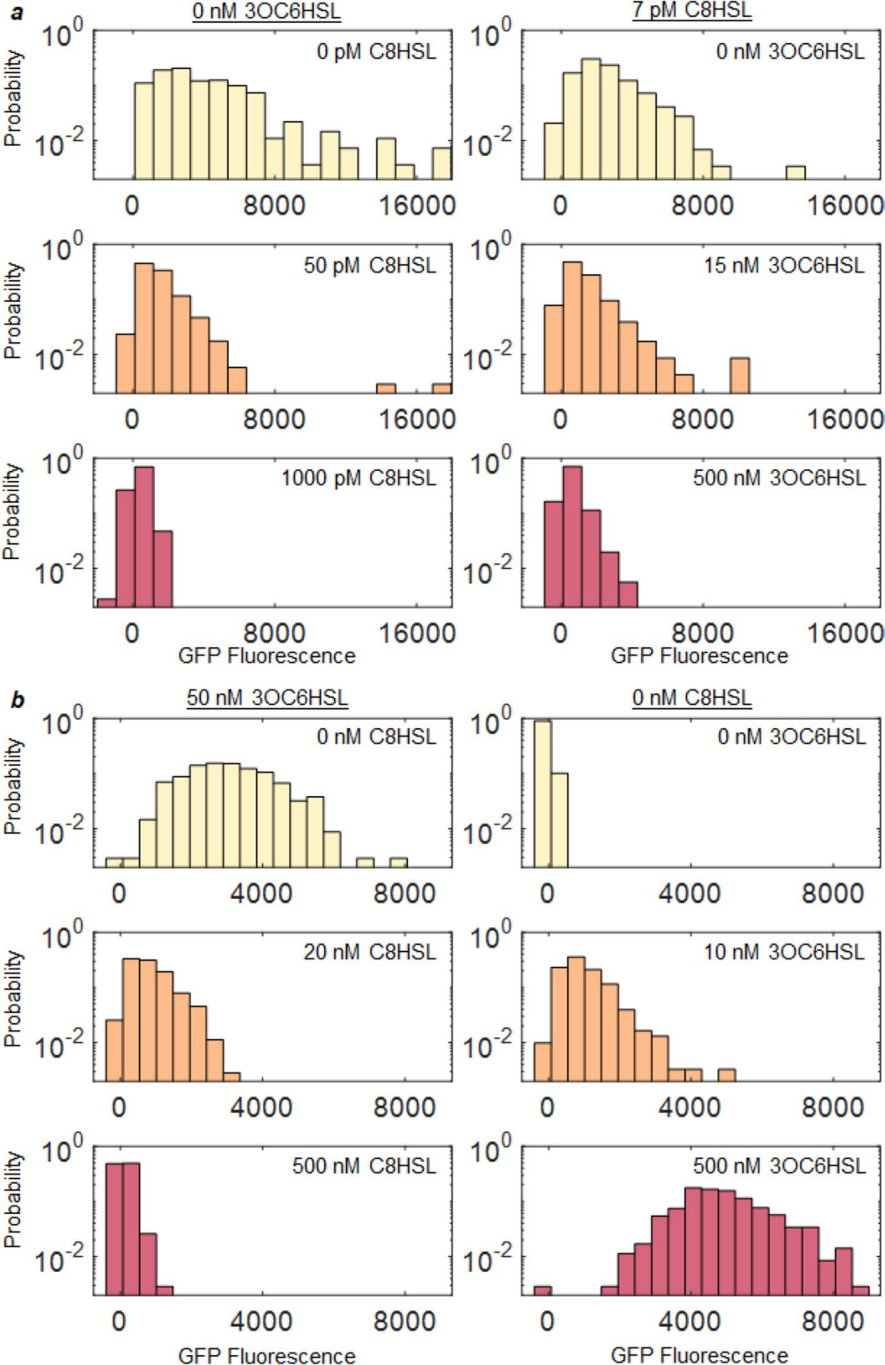
Combinations of HSL inputs shift the statistical distribution of P*qrr* and P*lux* activity in a population of *V. fischeri*. **(a)** Activity of *qrr* reporter measured in individual cells at different C8HSL concentrations with 3OC6HSL held fixed (left panels) or C8HSL fixed (right panels). **(b)** Activity of *lux* reporter at different C8HSL concentrations with 3OC6HSL held fixed (left panels) and C8HSL fixed (right panels). Each histogram summarizes the GFP reporter fluorescence measured for ~ 400 individual cells.

To study how the statistical distribution of *qrr* and *lux* reporter activity responds to combinations of the 3OC6HSL and C8HSL autoinducers, we measured the reporter fluorescence of individual cells immobilized on agarose pads. A histogram of individual cell fluorescence levels was collected for each combination of HSL concentrations. Figure 3 shows representative histograms for *qrr* (Fig. 3a) and *lux* (Fig. 3b). They are generally skewed or asymmetric, with both mean and variance responding to C8HSL and 3OC6HSL. Both HSLs alter the histograms for both reporters. As a result each reporter can exhibit a variety of possible distributions, depending on the HSL combination provided. Supplemental Figures S3 and S4 show the full set of individual cell fluorescence histograms collected.

The individual cell fluorescence measurements collected at each pair of HSL concentrations were first represented by a 20-bin histogram: Each histogram maps to one point in a transformed (20-dimensional) coordinate system where all possible histograms reside on the surface of the unit sphere (*Methods*). We then used principal component analysis (PCA) to perform a sensitive and model-free evaluation of these changes in the histograms. PCA provides a simplified representation of the histograms in a small number of dimensions to facilitate visualization and comparison of the histograms. It accurately characterizes how the histograms change under different experimental conditions without requiring assumptions about the underlying nature of those histograms (e.g. normal distributions, Poisson, etc.). It is therefore ideally suited to characterizing systems such as AinS/R and LuxI/R which exhibit a very complex and noisy response to their input variables (the HSLs). We performed PCA on the set of points obtained for each reporter.

**Figure 4:**
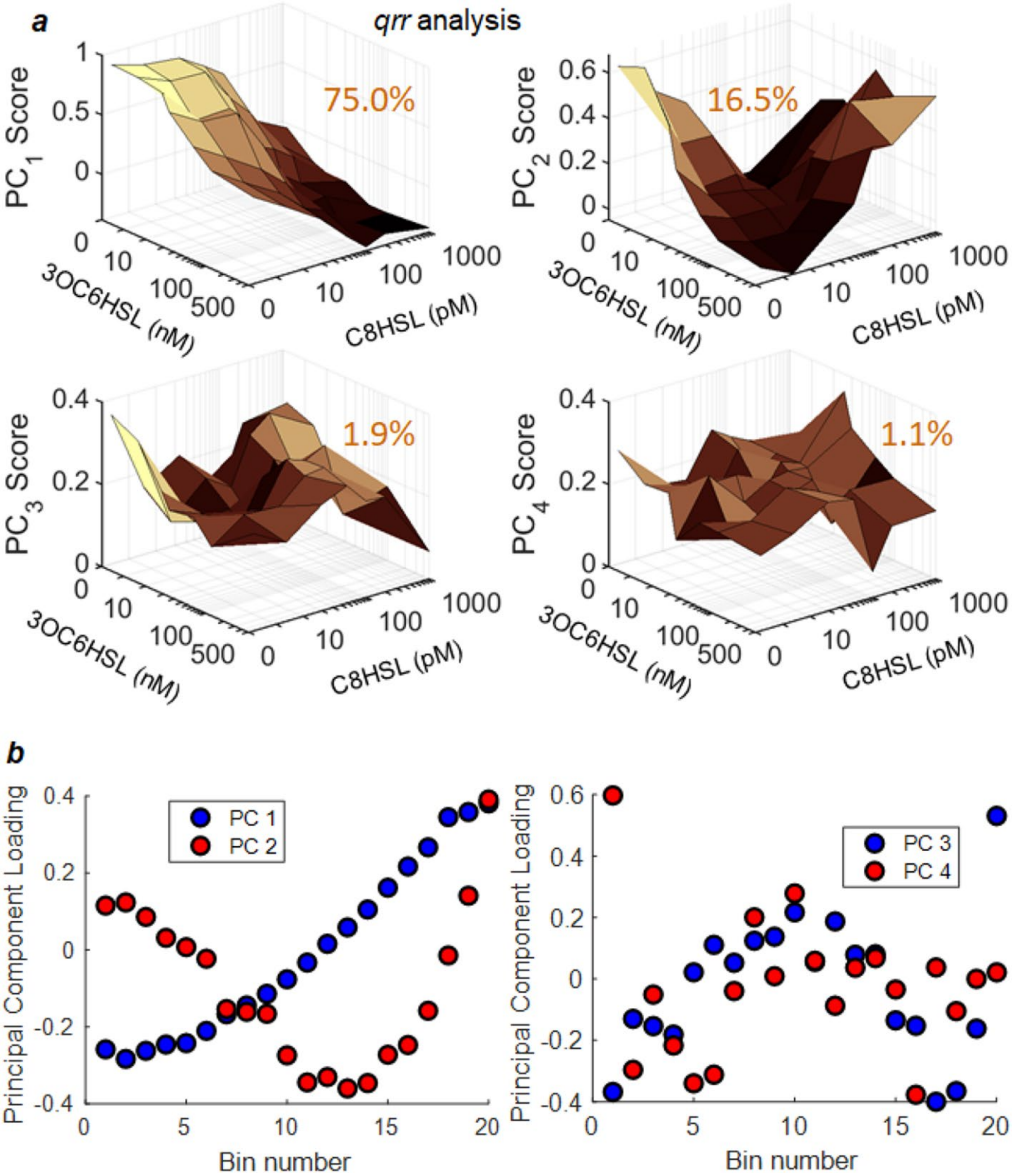
Principal component analysis (PCA) of the set of *qrr* reporter fluorescence histograms. Under each of 49 combinations of C8HSL and 3OC6HSL concentrations, the fluorescence measurements for ~ 400 individual cells were histogrammed into 20 bins and a square root transformation was applied to the bin heights (*Methods*). The PCA of the complete set of histograms then provides **(a)** scores for each principal component as a function of HSL input condition, and **(b)** loadings associated with each PCA component. The loadings describe the fluorescence-dependence described by each PCA component, and the scores describe the HSL dependence of that component. The percentage of data variance explained by each PCA component is indicated on the panel. The first four PCA components only are shown; these together account for 94.5% of the variance in the dataset (see text). HSL axes in **(a)** switch from linear to logarithmic at 10 concentration units^48^.

**Figure 5:**
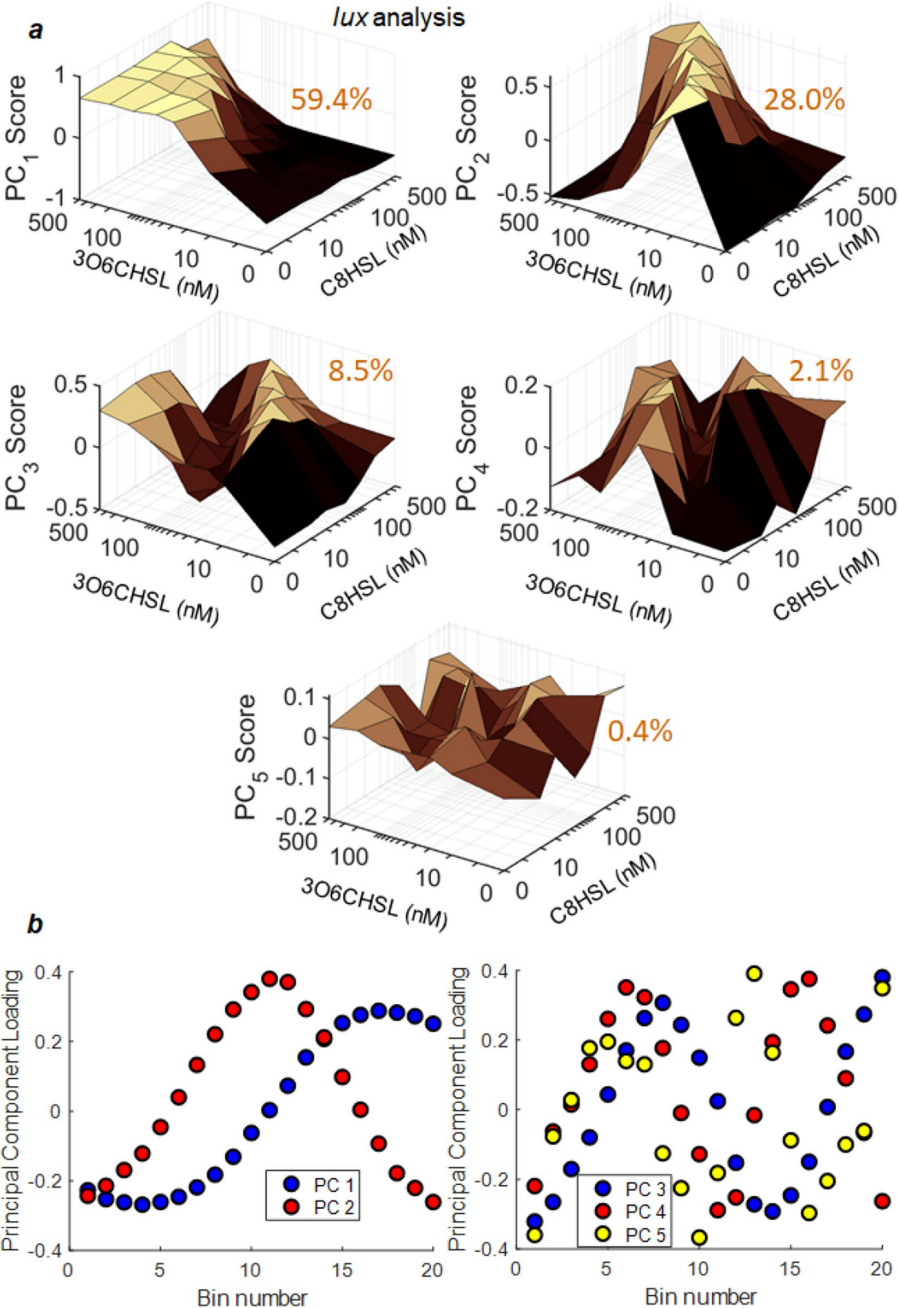
Principal component analysis (PCA) of the set of *lux* reporter fluorescence histograms, collected under 56 combinations of C8HSL and 3OC6HSL concentrations. The same analysis was performed as for the *qrr* reporter data. The PCA of the complete set of histograms provides **(a)** scores for each principal component as a function of HSL input condition, and **(b)** loadings associated with each PCA component. The percentage of data variance explained by each PCA component is indicated on the panel. For the *lux* data, the first five PCA components only are shown; these together account for 98.4% of the variance in the dataset (see text).

Figures 4 and 5 show the results for the *qrr* and *lux* reporter respectively. PCA produces scores (Figs. 4a, 5a) that describe the HSL dependence of each principal component, and loadings (Figs. 4b, 5b) that express those principal components as a set of underlying constituent histograms. For both reporters the 4–5 leading principal components are shown, but the first 1–2 components strongly dominate: For *qrr*, the first five principal components account for 75.0%, 16.5%, 1.9%, 1.1% and 0.8% of the variance in the data respectively. For *lux* the first five components account for 59.4%, 28.0%, 8.5%, 2.1% and 0.4% of the variance respectively. As a result, although the full histograms of individual cell fluorescence contain 20 bins, more than 90% of the changes in the histograms can be accurately described using only the low-dimensional representation contained in the two or three leading principal components.

**Figure 6:**
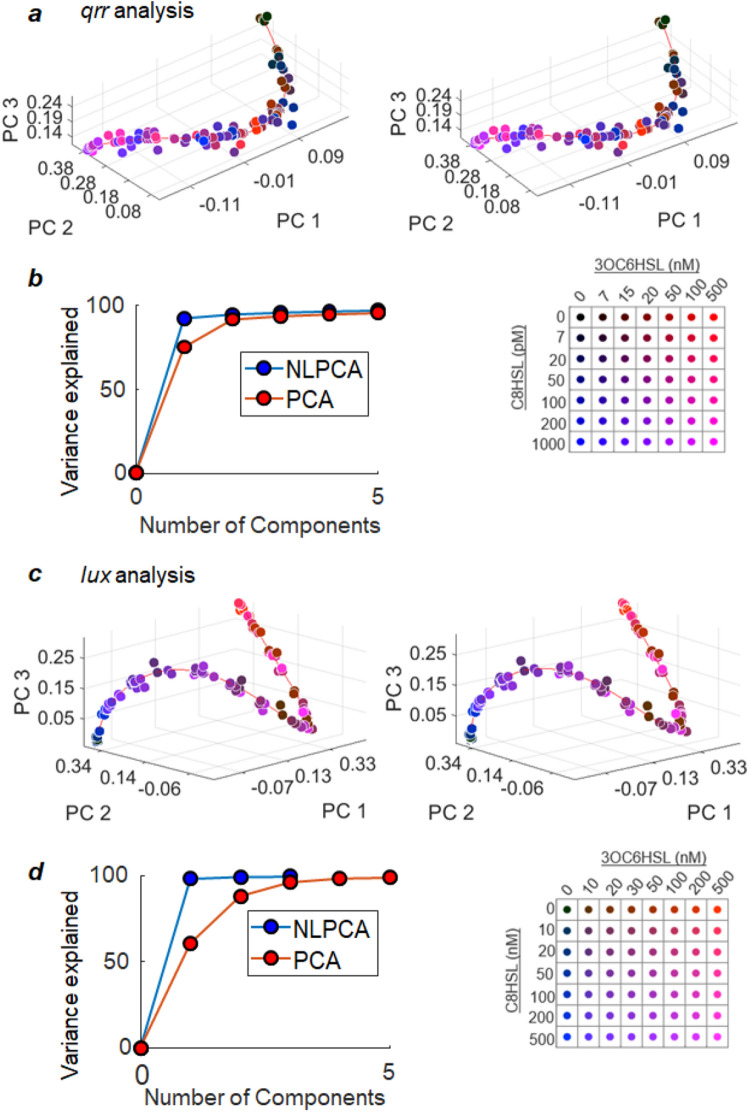
Low dimensional representation of the *qrr* and *lux* histograms. The set of PCA scores associated with each HSL concentration condition (Figs. 4a and 5a) defines a point in the multidimensional coordinate space of the principal components PC1, PC2, etc. The stereogram in **(a)** represents all such points for the *qrr* data in the three dimensional space of PC1-PC3. HSL conditions for each point are color-encoded using the key shown beneath **(a)**. The NLPCA analysis (“Methods”) further identifies a nonlinear coordinate, corresponding to the solid red curve in **(a)**, which threads the *qrr* data. As shown in **(b)**, this NLPCA coordinate is able to capture 92.2% of the variance in the full dataset. The stereogram in **(c)** similarly represents the points for *lux* in the three dimensional space of PC1-PC3. The solid red curve represents a single, one-dimensional nonlinear coordinate obtained by NLPCA. This coordinate threads the *lux* data and **(d)** captures 97.1% of the variance in the data.

For each reporter, the histogram of individual cell response for each HSL condition can then be displayed as one point in the low-dimensional space that corresponds to the three leading (linear) PCA components for that reporter. Figure 6a shows this representation for *qrr* as a stereogram: the points can be seen to lie along a twisted curve that is embedded in the three-dimensional space. From the upper right corner where no HSL is present, the introduction of HSL first causes a reduction in PC1 and PC3, followed by decrease and then increase in PC2. Comparison to Fig. 4b shows that these changes correspond to a shift of the *qrr* histogram toward lower activity, accompanied by a broadening and then narrowing of that histogram. Similarly for *lux*, Fig. 6c shows that introduction of HSL causes a nonuniform increase in PC1 and initial decrease in PC2; both PC2 and PC3 vary nonmonotonically. From Fig. 5b this corresponds to shift of the average *lux* activity towards larger values, accompanied by a nonlinear broadening and then narrowing of the histogram.

It is interesting that the *qrr* and *lux* data in Fig. 6a,c are not only well described by a small number of PCA dimensions, but within that low-dimensional space they appear to fall along a strongly nonlinear, nonmonotonic curve. To more carefully explore the dimensionality of this response, and to characterize its dependence on combinations of HSLs, we applied a hierarchical, nonlinear PCA (NLPCA) to our data. For a dataset that contains a large number of dimensions, NLPCA can identify a lower dimensional subspace that contains most of the useful information. Thus in this analysis where each histogram is a point in the space of the principal components, NLPCA identifies a smaller number of coordinates, related nonlinearly to the principal components, that describe the HSL-dependent variation in the histograms.

For both of the datasets of Figs. 4 and 5, the nonlinear PCA analysis identifies a single nonlinear component or coordinate, which is represented by the solid curve that threads the data in the stereograms of Fig. 6a,c. The positioning of the datapoints along this coordinate accounts for 92.2% of the variance in the *qrr* data, and 97.4% of the variance in the *lux* data. Thus a single NLPCA component is at least as effective as 3–4 linear PCA components in representing HSL-dependent changes in the histograms. Additional nonlinear PCA components did not provide significant improvement, as shown in Fig. 6b,d. The position along the solid curve in each stereogram is therefore a coordinate that efficiently describes how the statistical distribution of the entire population of cells changes in response to the input HSL conditions. The existence of this coordinate confirms that, regardless of the functional form of the full distribution of reporter responses, the distributions ultimately have a low dimensionality, and in fact are effectively one-dimensional for both reporters.

The color key in Fig. 6a, which is indexed to HSL conditions, also reveals that both HSLs must be provided in order to tune the *qrr* histogram over the full length of the solid curve. While the rightmost terminus of the arc (high PC1, PC3) corresponds to zero concentration of both HSLs, the leftmost terminus (low PC1, PC3) is only accessed when both HSLs are provided at high concentration. Saturating concentrations of either 3OC6HSL or C8HSL alone generally produce a response corresponding to the midpoint of the curve: Combinations of both HSLs allow *qrr* to exhibit some (low mean, small variance) distributions that are not accessible by addition of one HSL alone. A similar analysis of Fig. 6c shows that both HSLs are also needed in order to fine-tune the *lux* reporter response through its full range. The rightmost terminus of the solid curve in Fig. 6c (high PC3, PC1) is obtained when 3OC6HSL (but not C8HSL) is present at saturating concentrations, while the leftmost terminus (low PC1, high PC2) requires both HSLs be absent. The presence of C8HSL allows the system to explore *lux* behavior associated with the region of the curve near the left terminus. As with *qrr,* combinations of the two HSLs allow the histogram of *lux* reporter activity to take forms that do not occur in the presence of one HSL alone.

Because the histograms for both *qrr* and *lux* fall along one-dimensional trajectories in the principal component space, the shape of each reporter's statistical distribution is determined by the value of the NLPCA coordinate associated with each point along the trajectory. To provide a precise metric of the differences between histograms, we rescaled the single NLPCA coordinate (for each reporter) to give a new (single) coordinate denoted *S,* which lies in the range 0 → 1 and is a linear measure of distance in the principal component space. That is, equal increments in S_*qrr*_ or S_*lux*_ correspond to equal distances in the space of Fig. 6a or c respectively, and therefore equal chord distances between the underlying histograms. In this sense the difference in (for example) S_*qrr*_ for any two *qrr* histograms is a measure of the similarity or difference between those histograms.

Supplemental Figure S8 shows the histograms that correspond to some specific values of the S_*qrr*_ and S_*lux*_ coordinates. Supplemental Figure S9 shows how the mean, variance and related properties of these histograms depend on the *S* coordinate (S_*qrr*_ or S_*lux*_) for each reporter. These figures show that although the histograms are shaped through tuning of a single coordinate, they do not resemble common single parameter distributions such as Poisson or exponential, and they are poorly fit by other familiar distributions such as the gamma distribution.

**Figure 7:**
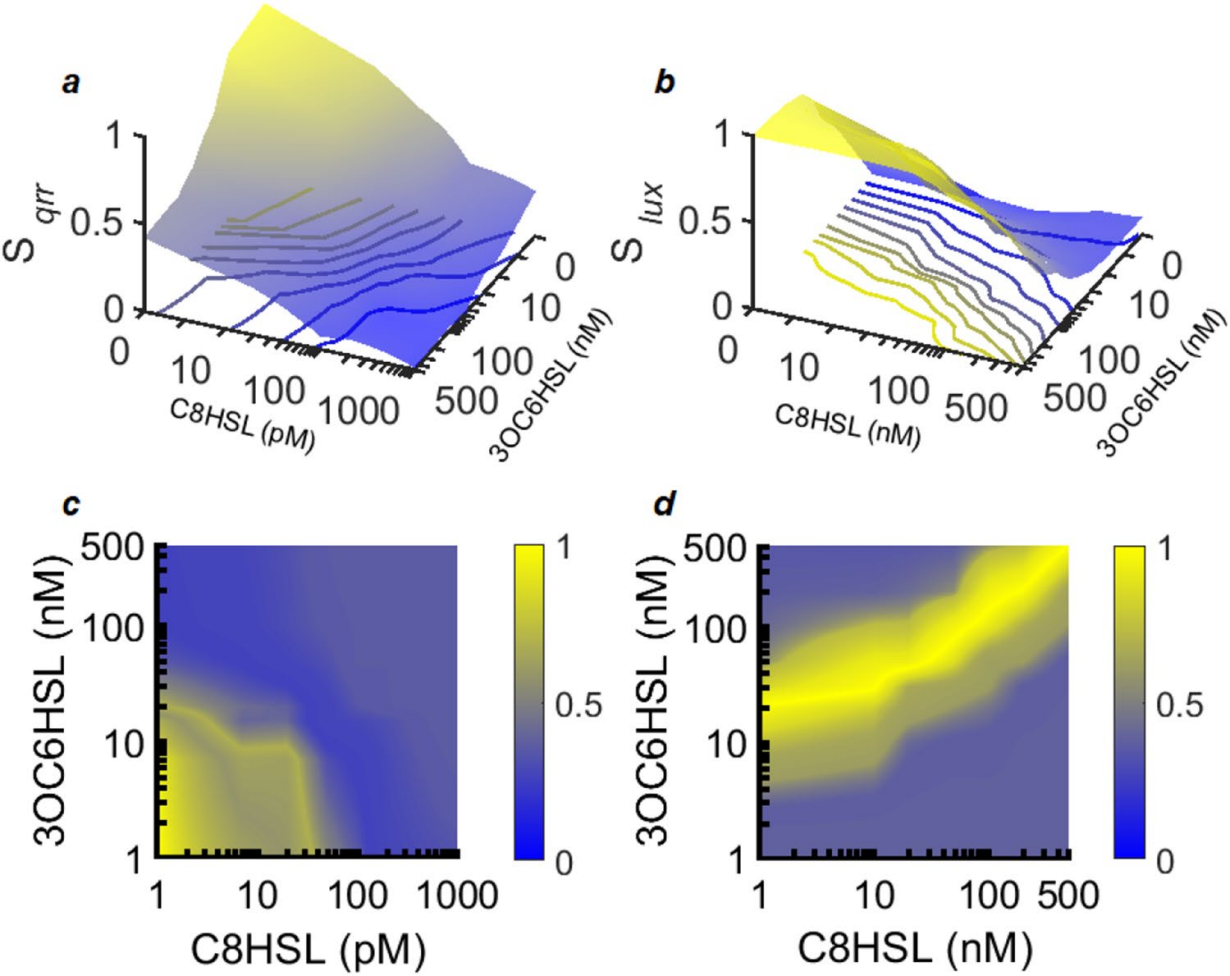
HSL-dependent response of the AinS/R and LuxI/R circuits is expressed in terms of the NLPCA coordinates *S*_*qrr*_ and *S*_*lux*_. These coordinates correspond to position (arc length) along the solid curve in Fig. 6a (for *qrr*) or Fig. 6c (for *lux*), where they capture > 92–97% of the HSL-dependence of the statistical distributions of individual cell behavior. *S*_*qrr*_ and *S*_*lux*_ are scaled to lie in the range 0 → 1 and are plotted with respect to HSL concentrations for **(a)** *qrr* and **(b)** *lux.* The color maps in **(c)** and **(d)** show the probability distributions for C8HSL and 3OC6HSL concentrations that would lead to flat probability distributions for **(c)** S_*qrr*_ and **(d)** S_*lux*_. That is, they show the HSL input probabilities for which all values of the NLPCA coordinate *S* would be equally probable. They indicate the HSL concentrations at which the S*qrr* and S*lux* surfaces in **(a,b)** have steepest slope and are changing most rapidly.

Figure 7 shows a map of the coordinates S_*qrr*_ and S_*lux*_, for *qrr* and *lux* respectively, versus the two HSL concentrations. Unlike plots of mean reporter activity (Fig. 2), these plots implicitly take account of cell-to-cell variability, contain no assumptions about the shape of the individual-cell fluorescence distribution, and therefore capture all significant changes in the individual cell response distribution as environmental inputs change.

Although the S_*qrr*_ and S_*lux*_ surfaces change rather little over some wide ranges of HSL conditions, there do exist HSL conditions for which the S_*qrr*_ and S_*lux*_ surfaces are steep, indicating more rapid changes in the underlying histograms when HSL concentrations change. A sensing pathway can be said to transmit the most information about its inputs under conditions where the output is most sensitive to the inputs^38^. Accordingly, we can use these surfaces to estimate the HSL conditions for which the statistical distributions for *qrr* and *lux* are most informative about changes in the environmental conditions experienced by the population. To find these conditions we evaluated the (prior) probability distribution of HSL concentrations for which the probability distribution for either S_*qrr*_ or S_*lux*_ would be as flat or uniform as possible; this approach maximizes the mutual information between the HSL concentrations and the output distribution. The maps in Fig. 7c,d show these probability distributions, where the slanted contour lines result from crosstalk between the two HSLs. They show that the *lux* histogram is most sensitive to changes in HSL when conditions lie in a zone that divides the contour plane diagonally, separating the region of mostly high 3OC6HSL/low C8HSL from the region of mostly low 3OC6HSL/high C8HSL. For *qrr* there is more than one sensitive zone; however, the *qrr* activity histogram is especially sensitive to HSL inputs when C8HSL concentrations are below ~ 30 pM and 3OC6HSL is below ~ 20 nM.

### HSL accumulation during growth

Mapping the input (HSL concentrations) and output (*S*_*qrr*_ and *S*_*lux*_) spaces of the LuxI/R and AinR/S systems reveals the HSL concentrations that are mostly likely to trigger these two branches of the sensing system. We can then ask what regions of the input/output space are actually explored during growth of a *V. fischeri* culture, and what transitions in the sensing system are triggered as a result. To determine the HSL conditions experienced during growth, we measured the accumulation of C8HSL and 3OC6HSL during growth of an in vitro culture of our background strain, ES114. We extracted growth medium periodically from an ES114 culture as it grew to OD (600 nm) = 1.5 and then used two HSL-sensing strains to test the C8HSL and 3OC6HSL concentrations present in those samples (“Methods”). Given the nanomolar HSL sensitivity of the sensing strains, this experiment generated an approximate measure of the time course of rising C8HSL and 3OC6HSL levels during culture growth.

**Figure 8:**
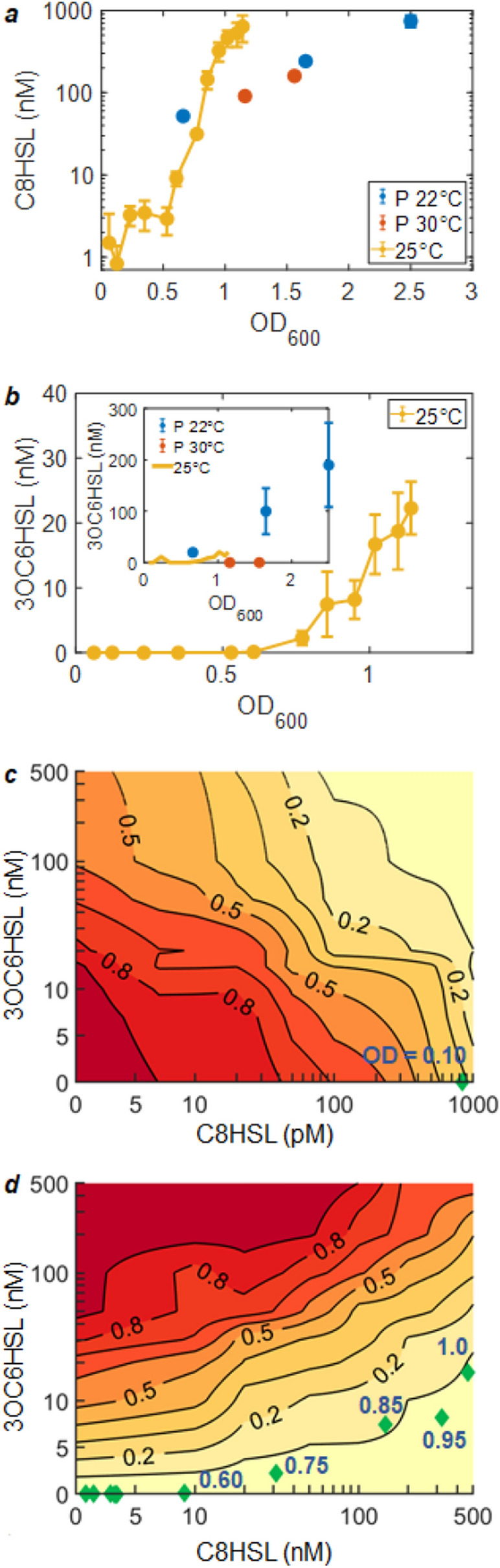
3OC6HSL and C8HSL concentrations attained during growth of an ES114 culture in vitro at 25 ^∘^C. Concentrations of **(a)** C8HSL and **(b)** 3OC6HSL measured during growth to OD 1.2. Data from^7^ for growth at 22 ^∘^C and 35 ^∘^C are shown for comparison. The resulting trajectories in HSL concentration are shown in **(c,d)**, overlaid onto contour plots of **(c)** *S*_*qrr*_ and **(d)** *S*_*lux*_, to show what range of distributions of *qrr* and *lux* activity are encountered as the optical density rises during growth. OD values associated with the points shown as green diamonds are indicated by the point labels.

The data show, consistent with several previous reports^7,18^, that *V. fischeri* ES114 produces C8HSL prodigiously during growth in vitro, but its production of 3OC6HSL is much more limited^7,27,39^. As shown in Fig. 8a, C8HSL already exceeds ~ 1 nM before OD 0.5, and exceeds 200 nM by OD 1. By contrast, 3OC6HSL did not reach ~ 10–20 nM until OD 1, Fig. 8b. Accordingly, *qrr* expression is expected to transition from *ON* to *OFF* very early in growth, and reach a strongly *OFF* state virtually as soon as the culture achieves a population density of OD ~ 0.1, Fig. 8c. By contrast the full activation of *lux* does not occur at the population densities studied here, owing to underproduction of 3OC6HSL^13,27^, Fig. 8d. In fact, the slow accumulation of 3OC6HSL indicates that the activation of *lux* remains at a rather constant low level during virtually the entire growth curve: for *lux* the HSL concentrations from OD near zero until OD ~ 1 rather closely follow a contour line of *S*_*lux*_ ~ 0.1. The adherence to this contour indicates not only that *lux* remains mostly OFF through most of the in vitro growth curve, but that even the statistical distribution of individual cell *lux* activity undergoes little or no change during this time.

## Discussion

Bacterial pheromone sensing systems typically have a complicated structure that links multiple signals and their detection pathways through crosstalk and regulatory feedback. This complexity raises questions of what information these systems gain by detecting more than one autoinducer species, what environmental changes they are tuned to detect, and how interactions between incoming signals modify the range of responses of each sensing pathway.

Single cell measurements have shown that phenotypic heterogeneity is substantial in these sensing systems and that it may play an important role in pheromone sensing strategies^40^. The analysis of the information flow in a multiple autoinducer system should address how the crosstalking signals and receptors affect the statistical distribution of individual cell response, rather than just average behaviors. Although combinations of multiple receptor channels can tune phenotypic heterogeneity^24^, the detection of more than one HSL does not itself increase the information available to an individual cell, as might be expected if the multiple signals served a coincidence detection function^41^. However, because the output of a pathway is statistical in character, it can be difficult to visualize and quantify the effects of combinations of autoinducer signals on sensing function.

The LuxI/R and AinS/R branches of the *V. fischeri* pheromone sensing pathway are linked by crosstalk in the form of noncognate HSL-receptor interactions, upstream regulation of LuxR via LitR, and transcriptional repression of *qrr* (in addition to feedback effects). Here we have used dimension-reduction to explore and visualize how crosstalk modifies the statistical distribution of output of each branch. The activity of a *lux* reporter provided an experimental readout for the LuxI/R system. Because *qrr* is directly driven by the LuxO/LuxU phosphorelay and is the immediate regulator of *litR*^28^, we used *qrr* promoter activity as a readout of the response of the AinR/S-controlled pathway.

We used PCA to perform a model-free, dimension-reduction of the responses of individual cells. This method characterizes changes in the *qrr* and *lux* reporter distributions without making assumptions about what statistical measures, such as mean or variance, have greatest significance in the population-wide response of cells. The analysis shows that for both the AinR/S and LuxI/R pathways, the full set of statistical distributions of output activity can be accurately represented in a low-dimensional space; in fact they can be represented as points embedded in a low-dimensional space, where these points fall closely along a simple threading curve. The curve takes a more complex form for *lux*, where it is nonmonotonic in each of the principal component dimensions, than for *qrr*.

Distributions of individual cell behavior can be shaped by many mechanisms, and both the *lux* and *qrr* branches of the system are presumably subject to intrinsic and extrinsic noise as well as crosstalk, which may combine in various ways to generate the observed distributions. The qualitative difference between the *qrr* and *lux* trajectories in Fig. 6a,c may reflect the fact that HSLs impact *lux* activity through both a regulatory/transcriptional pathway and by direct binding to the LuxR receptor. That is, a higher level of *lux* activity could be achieved both by higher 3OC6HSL levels binding to existing LuxR—an effect driven by the 3OC6HSL inputs—and by higher LitR levels leading to increased production of LuxR—an effect driven largely by C8HSL. Both of these effects would be expected to increase average *lux* activity, but as they involve different LuxR copy numbers they would be expected to lead to differences in *lux* variance. That is, variance in *lux* activity can be seen as having both intrinsic (due to 3OC6HSL-LuxR interactions) and extrinsic (due to LitR and LuxR levels) origins. At the same time, the effect of C8HSL on LitR (and hence LuxR) can be ameliorated to some extent by the crosstalking effects of 3OC6HSL on *qrr.* Thus the architecture of the system appears to allow the mean and variance of *lux* activity to be manipulated through multiple, but not entirely independent, routes.

Given that the HSLs modulate both *qrr* and *lux* through such interlocked mechanisms, it is surprising that the full range of population-wide responses to varying two signal inputs tunes the output in an essentially one-parameter fashion. An interesting line of further study could be to explore the robustness of this result under perturbation of the system, using mutant strains. One could investigate for example whether the same general family of distributions represented in Fig. 6 are still present in mutant strains where some of these crosstalk mechanisms are disrupted, such as by constitutive expression of *luxR*.

In some other, well studied systems where the crosstalk between autoinducer signals is very pronounced it has been possible to identify distinct roles for individual signals. For example, *Vibrio harveyi* detects three signals (including two HSLs) in a mostly parallel mode where information from the three dedicated receptors merges in a common phosphorylation cascade. The three signals have been interpreted as intraspecies, intragenus, and interspecies signals, respectively^42^. They accumulate at different rates during the growth curve^11^ and drive the outputs with different efficiency, so that the regulated phenotypes are expressed with different profiles during the growth curve. Similarly in *Vibrio cholerae* the intragenus and interspecies autoinducers appear to act jointly in the decision to disperse from a biofilm^43^. Consequently those multi-signal circuits may provide information about timing and species composition as a microbial community develops.

The *V. fischeri* pathway has a more sequential structure in which the AinS/R and LuxI/R branches have distinct regulatory outputs. This has led to its interpretation as a two-stage mechanism that serves a timing function, with colonization behaviors triggering earlier due to AinS/R and bioluminescence induced later through LuxI/R^13,44^. Nevertheless the multiple mechanisms of crosstalk between the two branches raises the question of how interactions between the HSLs tune the overall response and sensitivity. Our finding that two dimensions of input stimulus elicit only a one-dimensional output from each branch could mean that the combinations of crosstalking autoinducers do not change the possible responses of the sensing system beyond what is observed with a single autoinducer. However that appears not to be the case. Instead we find (for both *lux* and *qrr*) that the crosstalking (second) autoinducer drives the system's representative point (or the value of *S*_*qr*r_ or *S*_*lux*_) through portions of the response curve (Fig. 6a,c) that are not sampled when only the first autoinducer is present. In the case of *qrr*, mixtures of both HSLs allow the system to travel further along the one-dimensional curve than it would in the presence of only one HSL: The presence of 3OC6HSL forces *qrr* into a more strongly *OFF* state than occurs even at high C8HSL concentrations. In the case of *lux*, the presence of C8HSL allows the level of *lux* expression to be more finely tuned between ON and OFF states: C8HSL allows a more gradual, less all-or-nothing, switching of the population-wide response of *lux*.

The fact that *V. fischeri* senses two HSL autoinducers raises the question of whether the relative contributions of the two sensing pathways changes in a continuous fashion as HSL levels increase, allowing the two systems work together to control behavior at different stages of colonization^44^. The low-dimensionality of the response to HSLs simplifies the problem of determining when each pathway is maximally sensitive to the HSL inputs, and this may allow some insight into the functionality that is gained through the two HSLs. Our PCA analysis and our measurements of actual HSL concentrations together indicate that in ES114 both the *lux* and *qrr* outputs are mostly switched off during the overwhelming majority of the growth curve. The ES114 strain does not pass through either of the HSL regimes of high sensitivity except at the very earliest and latest stages of growth. Other than an early switching *OFF* of *qrr* (before OD = 0.10), and a very late switching *ON* of *luxI* (after OD = 1.0), there are few changes in either the *qrr* or *lux* histograms during the overwhelming majority of the growth curve in vitro.

Consequently, although the system could be described as having a timing function, it may be useful to distinguish between a clock, which continuously reports the passage of time, and an alarm, which does not. Aside from early switching of AinS/R, the joint output of the two branches appears insensitive to the changes in HSL concentrations that would signal progression of ES114 through various intermediate population densities. Without the crosstalking effect of C8HSL, the statistical distribution of *lux* activity would shift as OD increases, and so the behavior of *lux* would contain information about the culture density. In fact however, we find that crosstalk makes the response of *lux* surprisingly insensitive to the age or density of a growing culture, at least until OD > 1. In this sense crosstalk between the two HSLs provides a strong delay functionality to the AinS/R/LuxI/R system although it actually reduces the system's ability to detect or respond to intermediate population densities.

Finally we note that HSL production by different *V. fischeri* strains is highly variable^45^. In addition, the LuxR-HSL interactions are highly tunable through sequence variations in LuxR^31,46^, while the *luxI-luxR* intergenic region that regulates *lux* is highly divergent in comparisons between different *V. fischeri* strains^47^. Accordingly different strains should vary rather considerably, not only in the shape of the HSL sensitivity contours for their LuxI/R and AinS/R branches, but also in the trajectory with which they cross these contours during growth and colonization. It is an interesting question how this may allow *V. fischeri* strains other than ES114 to sense and respond to different combinations of environmental variables in optimal fashion.

## Supplementary Information

Supplementary Figures.

Supplementary Information 1.

Supplementary Information 2.

## Data Availability

The datasets generated and analyzed during the current study are available from the corresponding author on reasonable request.
