# Supplementary material for: Dimension-reduction simplifies the analysis of signal crosstalk in a bacterial quorum sensing pathway
Source: Sci Rep. 2021 Oct 5;11:19719. doi: 10.1038/s41598-021-99169-0 (PMC8492804; doi:10.1038/s41598-021-99169-0)
Supplement: Supplementary file 1 — Supplementary Figures. [file 41598_2021_99169_MOESM1_ESM.pdf]

Supplementary Information

Dimension-reduction simplifies the analysis of signal crosstalk in a bacterial quorum sensing pathway

Taylor Miller, Keval Patel, Coralis Rodriguez, Eric Stabb, Stephen Hagen

Contents

Figure S1 – Green and red fluorescence correlation in the *qrr* reporting strain 2
Figure S2 – Background correction for individual-cell GFP fluorescence 3
Figure S3 – Histograms of individual cell GFP fluorescence for *qrr* reporter....................................... 4
Figure S4 – Histograms of individual cell GFP fluorescence for *lux* reporter...................................... 5
Figure S5 – Histograms of *qrr* reporter activity for PCA 6
Figure S6 – Histograms of *lux* reporter activity for PCA.. 7
Figure S7 – GFP fluorescence intervals associated with histogram bins 8
Figure S8 - Fluorescence histograms at different values of the NLPCA coordinate 9
Figure S9 – Properties of the fluorescence histograms vs NLPCA coordinate.................................. 10

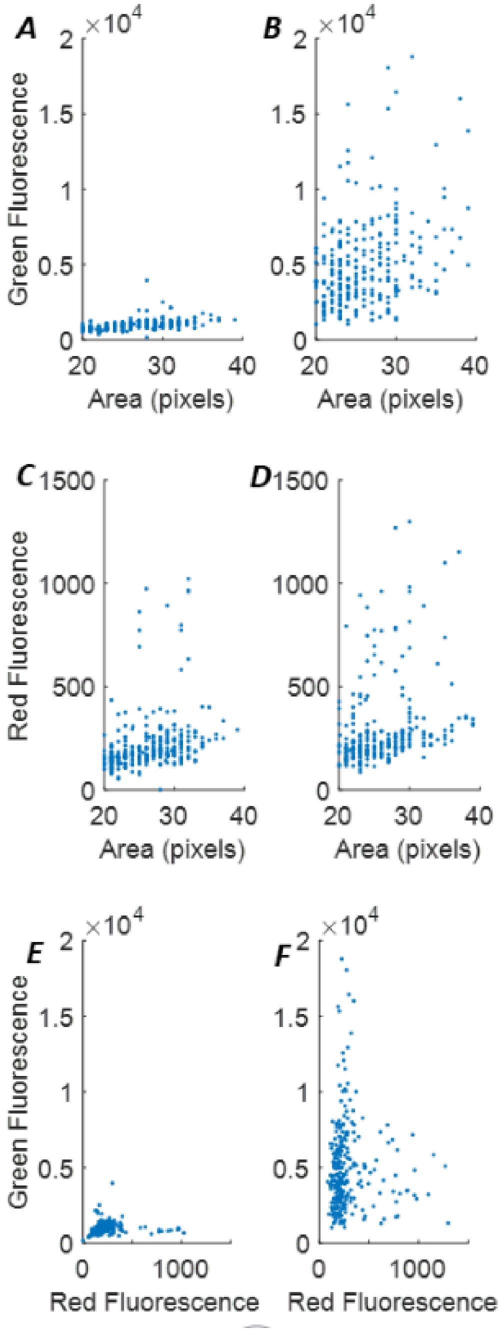

Figure S1 – Green and red fluorescence correlation in the *qrr* reporting strain

Scatter plots showing correlations between GFP, RFP and cell area in individual cells of the P*qrr-gfp* reporting strain, from fluorescence microscopy. (A),(C),(E) show correlations in media containing 500 nM 3OC6HSL + 1000 pM C8HSL where *qrr* reporter activity is low. (B),(D),(F) show cells from media lacking added HSL where reporter activity is high. (A) shows a weak, area-dependent baseline in the green fluorescence that is less apparent when *qrr* is activated in (B).

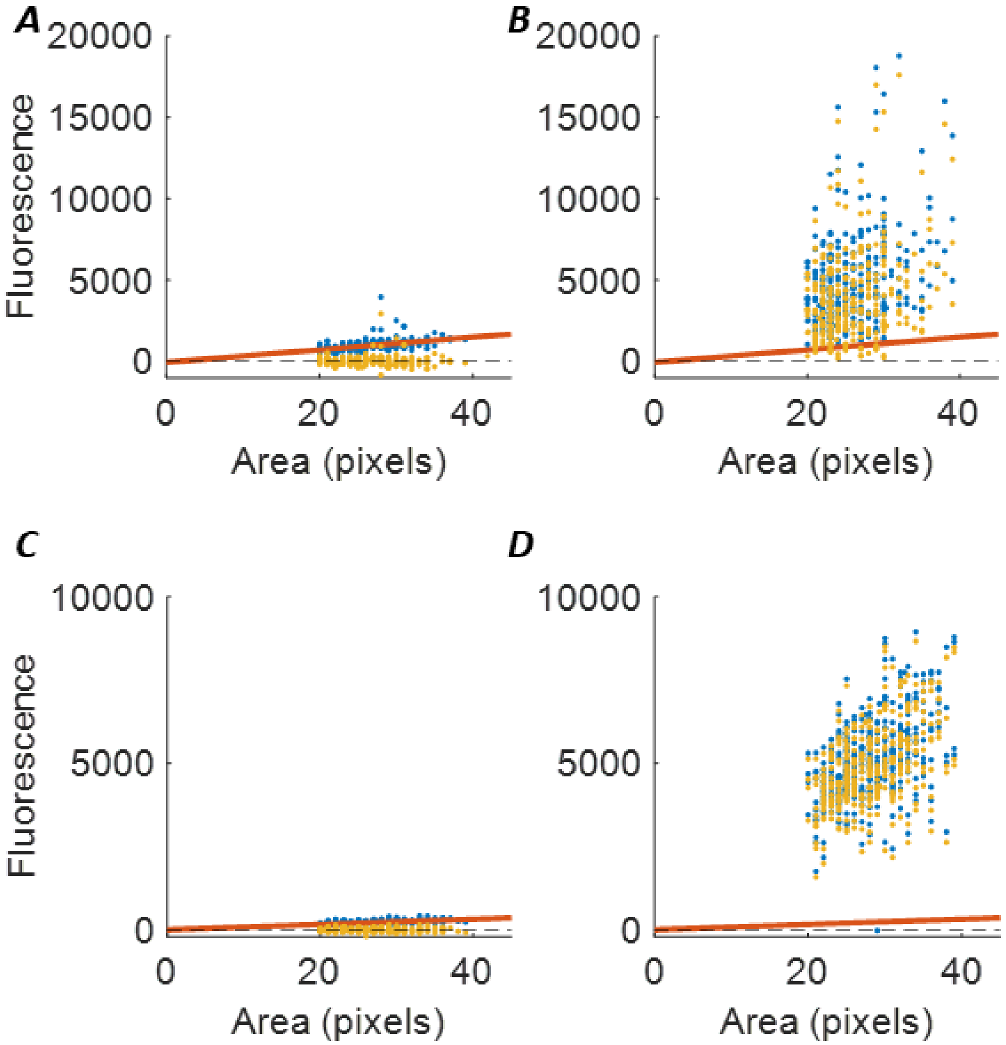

Figure S2 – Background correction for individual-cell GFP fluorescence

Measurements of individual-cell GFP fluorescence were corrected for a weak, cell-size dependent background as shown. In (A) the HSL condition of 500 nM 3OC6HSL + 1 nM C8HSL results in a very weak GFP signal for the *qrr* reporting strain. The fluorescence of individual cells (blue points) shows a weak trend with cell area, which is measured in pixels. This trend can be subtracted to give the corrected points (yellow). In the no-HSL condition (B), the GFP fluorescence is generally much greater and the area correction has a smaller impact on the data. For the *lux* reporting strain, the no-HSL condition in (C) results in weak fluorescence and the size-dependent trend is apparent (blue dots), and the correction leads to nearly zero average fluorescence (yellow dots). For the *lux* reporter strain under 500 nM 3OC6HSL (D) both the corrected and uncorrected fluorescence values are similar.

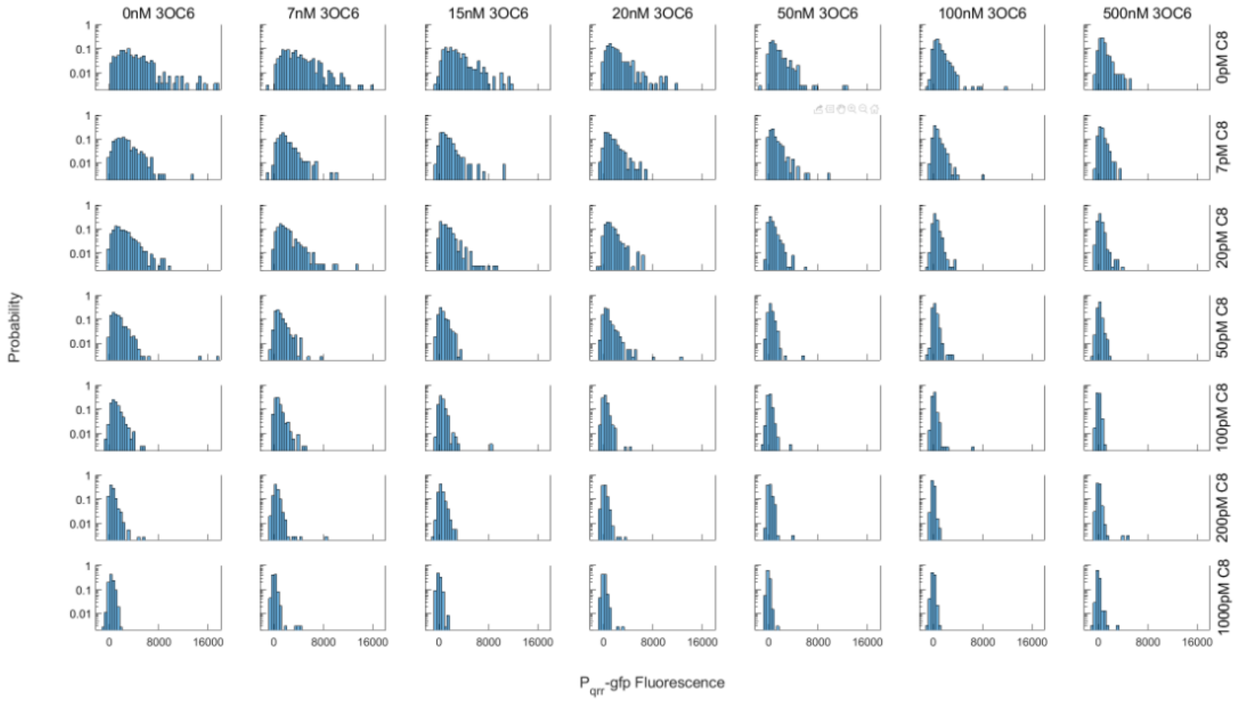

Figure S3 – Histograms of individual cell GFP fluorescence for *qrr* reporter

GFP fluorescence of individual cells carrying the *qrr* reporter was measured for approximately 400 cells at each of the 49 HSL input conditions shown above. The histograms are shown as probabilities on a logarithmic vertical scale.

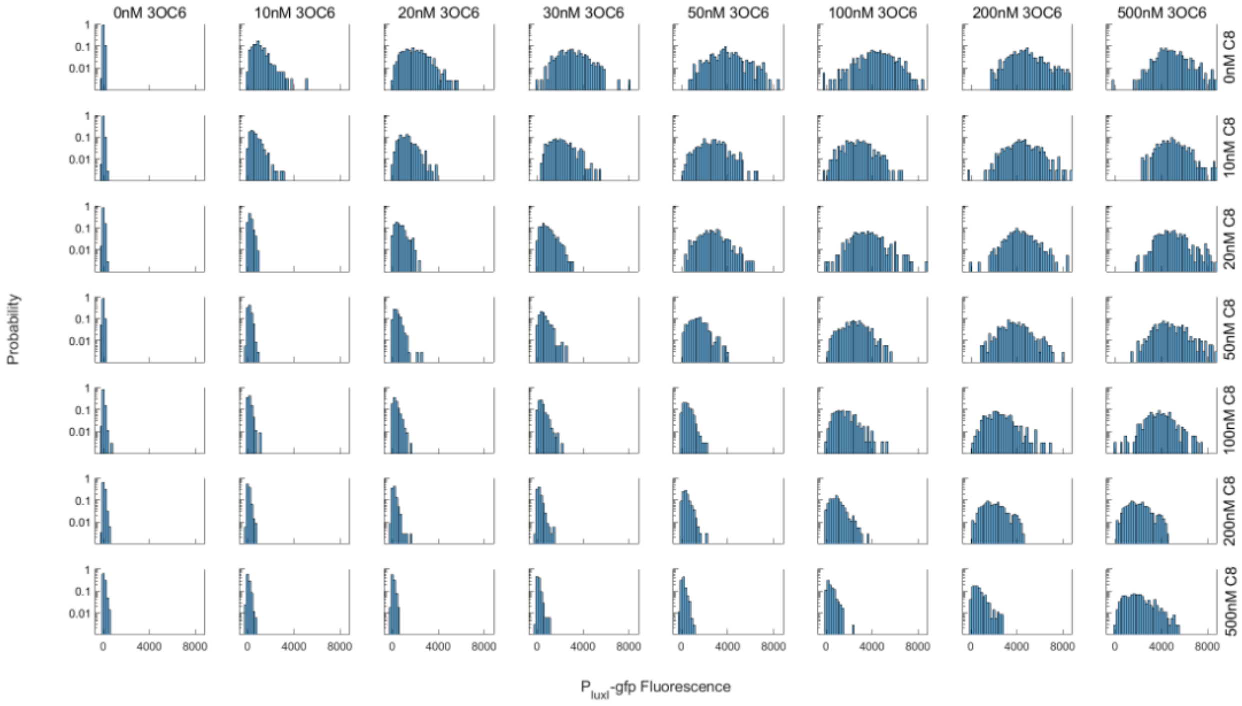

Figure S4 – Histograms of individual cell GFP fluorescence for *lux* reporter

GFP fluorescence of individual cells carrying the *lux* reporter was measured for approximately 400 cells at each of the 56 HSL input conditions shown above. The histograms are shown as probabilities on a logarithmic vertical scale.

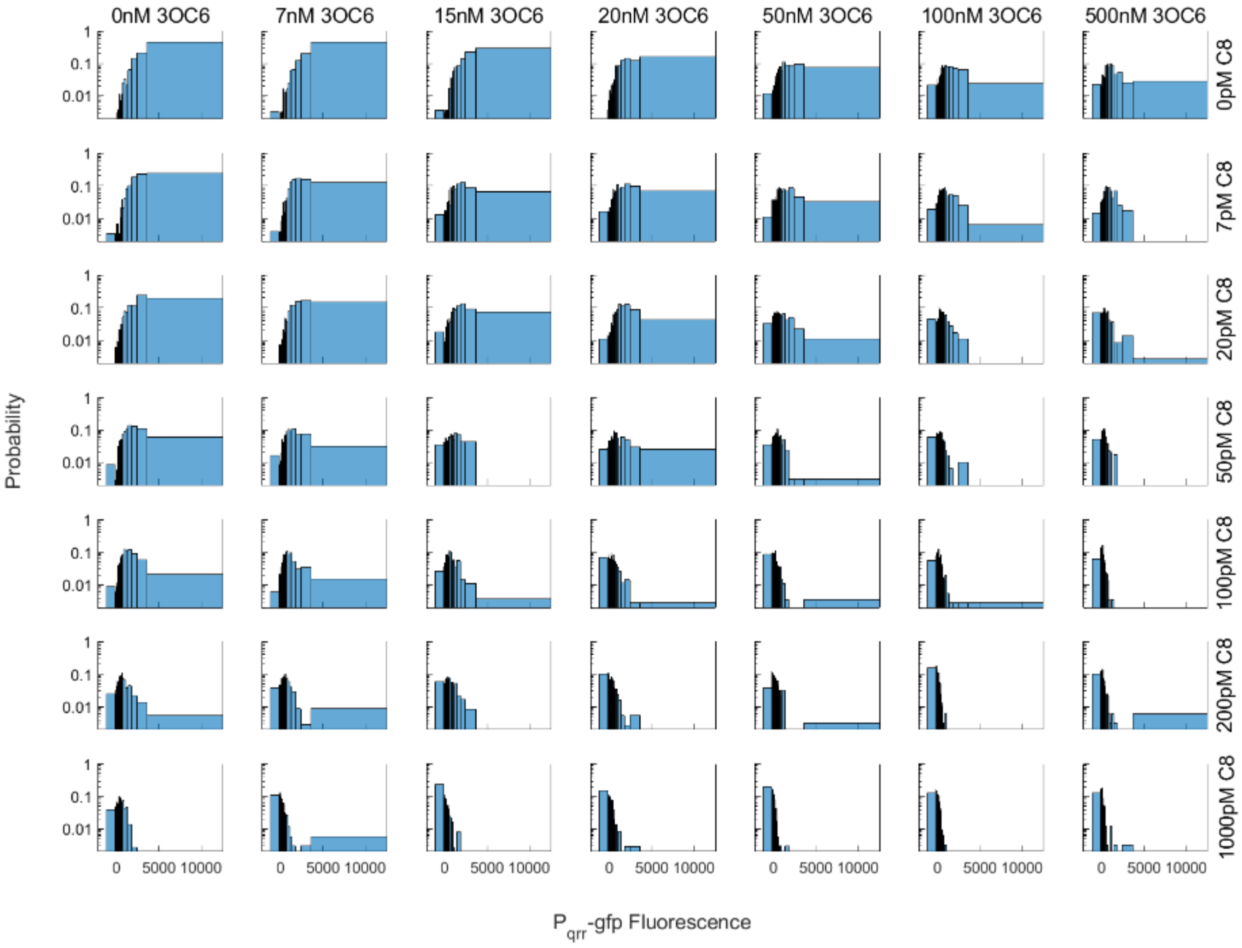

Figure S5 – Histograms of *qrr* reporter activity for PCA

For PCA analysis the *qrr* reporter fluorescence data were rebinned into the 20-bin histograms that are shown. Each histogram shows the probability that the GFP fluorescence of a cell under the indicated C8HSL and 3OC6HSL falls into the indicated bin. Bins are not equally spaced: bin widths were chosen so that in the aggregate of all individual cell fluorescence measurements under all HSL conditions, all bins contain the same number (797-798) of cells.

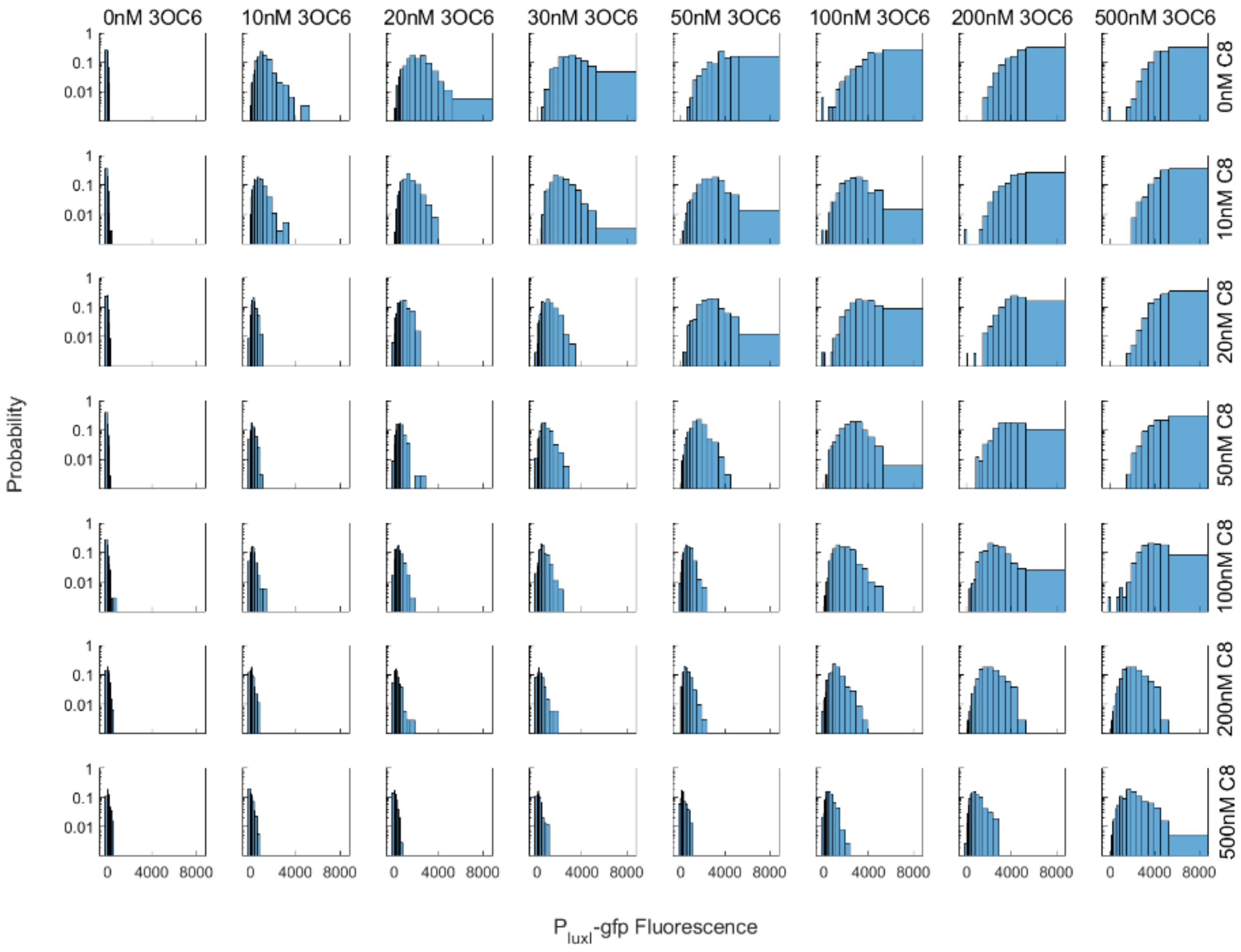

Figure S6 – Histograms of *lux* reporter activity for PCA

For PCA analysis the *lux* reporter fluorescence data were rebinned into the 20-bin histograms that are shown. Each histogram shows the probability that the GFP fluorescence of a cell under the indicated C8HSL and 3OC6HSL falls into the indicated bin. Bin widths were chosen so that in the aggregate of all individual cell fluorescence measurements under all HSL conditions, all bins contain the same number (964-965) of cells.

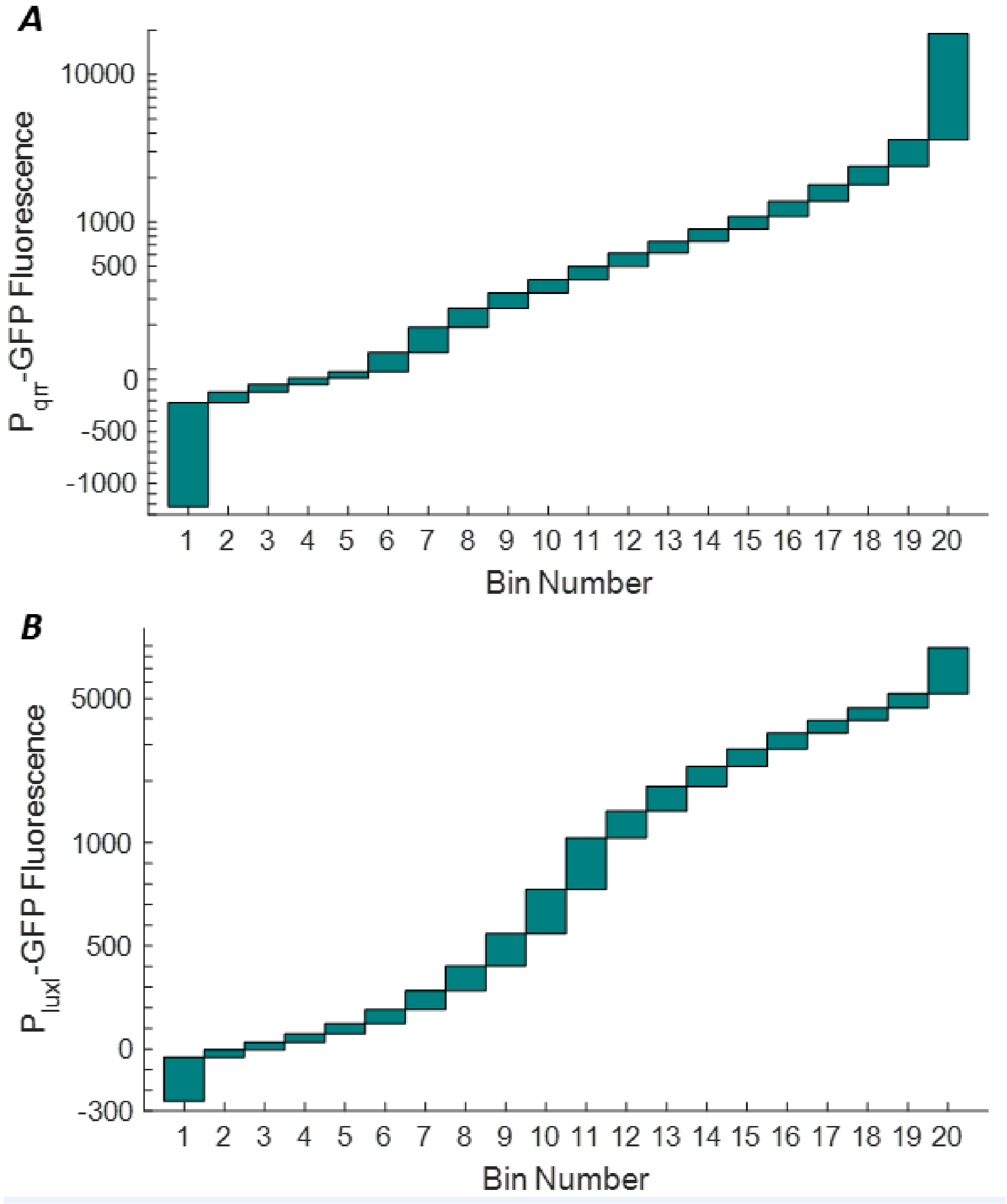

Figure S7 – GFP fluorescence intervals associated with histogram bins

The histogram bins used for PCA analysis of the (A) *qrr* and (B) *lux* reporter data are shown. The bins were selected so that in the aggregated dataset of all cells measured under all HSL conditions for a given reporter, an equal number of cells falls into each bin. In (A), the y-scale is linear below 100 and logarithmic above that value. In (B), the y-scale is linear below 1000 and logarithmic above that.

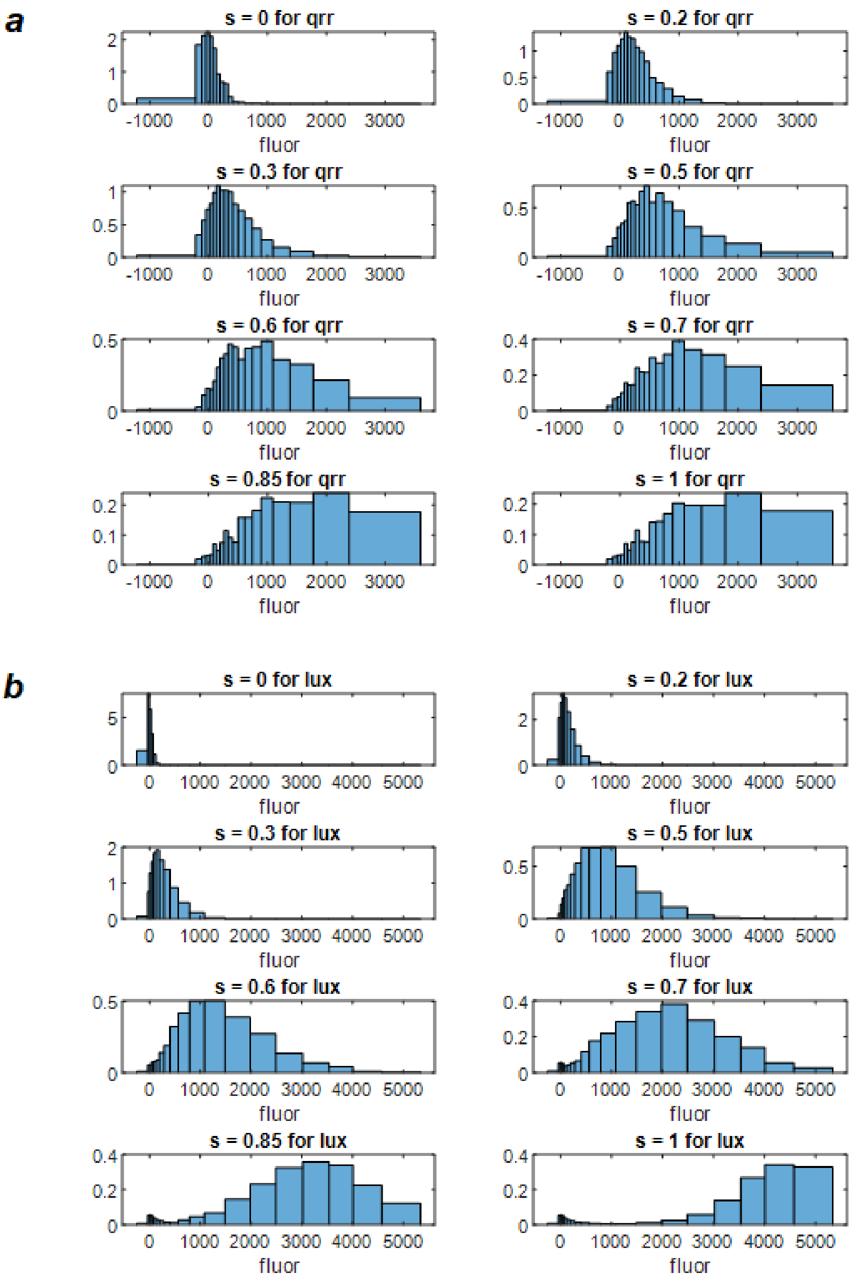

Figure S8 - Fluorescence histograms at different values of the NLPCA coordinate

Principal component analysis (PCA) is applied to the family of reporter fluorescence distributions obtained for each reporter under various HSL conditions. The value of s ($0 \leq s \leq 1$) defines a position along a one-dimensional trajectory that is embedded in the space of PC1, PC2, etc. and therefore completely defines the histogram of individual cell fluorescences that are obtained for one particular combination of HSL inputs. (The parameter s refers to s_{qrr} or s_{lux} for the *qrr* or *lux* reporter respectively.) For the (a) *qrr* reporter and (b) lux reporter data, the panels show the fluorescence histograms that correspond to s = 0, 0.2, 0.3, 0.5, 0.6, 0.7, 0.85 and 1.0.

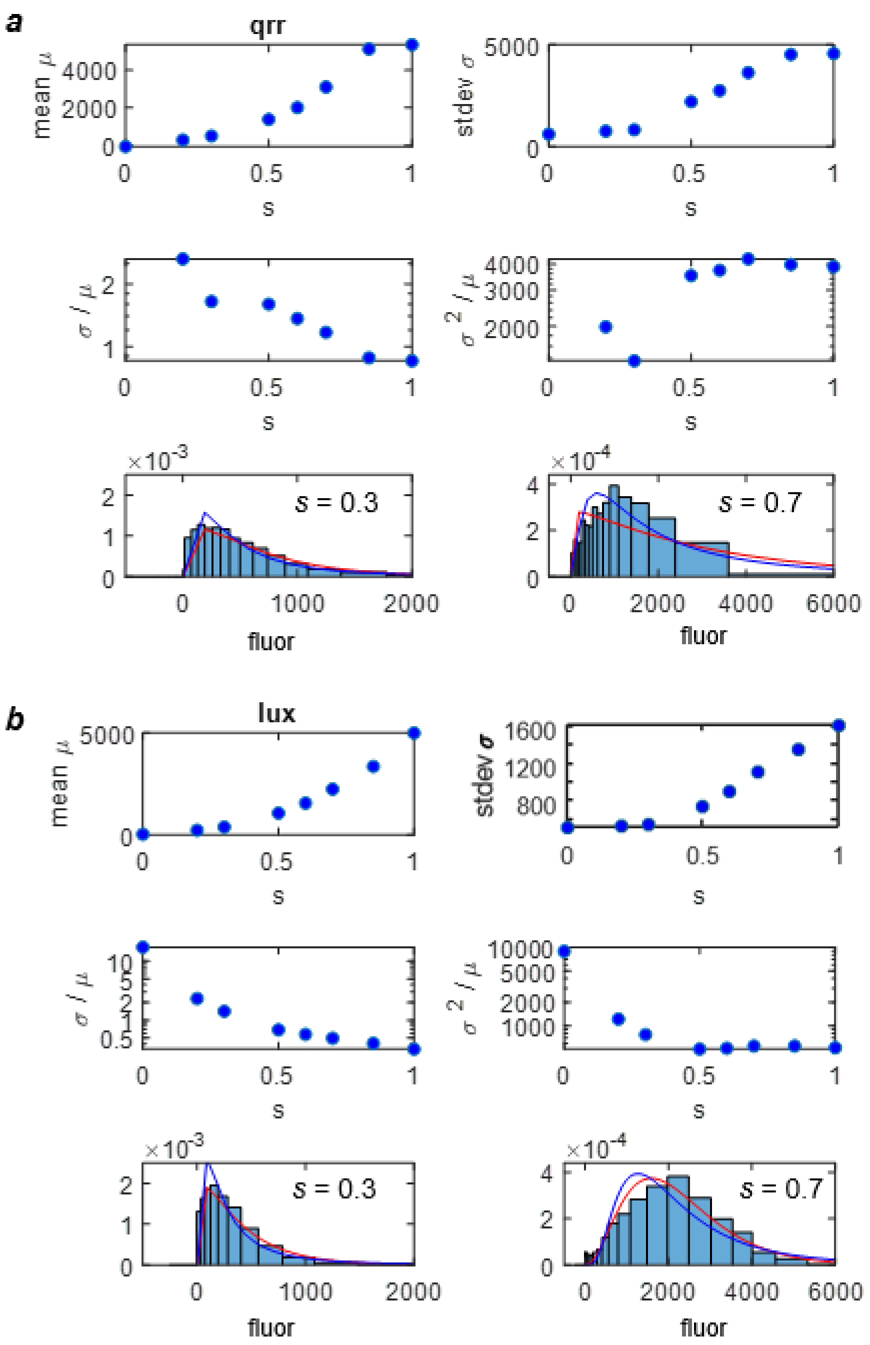

Figure S9 – Properties of the fluorescence histograms vs NLPCA coordinate

As each value of *s* defines a histogram of individual cell reporter fluorescences, the mean μ and variance σ^2 of the reporter fluorescence are functions of *s.* The panels show the dependence of μ, σ, σ^2, σ/μ and σ^2/μ on *s* for the (a) *qrr* and (b) *lux* reporters. For each reporter the reporter fluorescence histogram for *s* = 0.3 (left) and *s* = 0.7 (right) is fit to a gamma (red curve) and log normal (blue curve) distribution as shown.
